# Sequence features associated with the cleavage efficiency of CRISPR/Cas9 system

**DOI:** 10.1038/srep19675

**Published:** 2016-01-27

**Authors:** Xiaoxi Liu, Ayaka Homma, Jamasb Sayadi, Shu Yang, Jun Ohashi, Toru Takumi

**Affiliations:** 1 RIKEN Brain Science Institute, Wako, Saitama, Japan; 2 Harvard College, Cambridge, Massachusetts 02138, United States; 3 Department of Computer Science, University of British Columbia, Vancouver, Canada; 4 Department of Biological Sciences, Graduate School of Science, University of Tokyo, Bunkyo, Tokyo, Japan; 5 Core Research for Evolutional Science and Technology (CREST), Japan Science and Technology Agency (JST), 7 Gobancho, Chiyoda-ku, Tokyo 102-0076, Japan.

## Abstract

The CRISPR-Cas9 system has recently emerged as a versatile tool for biological and medical research. In this system, a single guide RNA (sgRNA) directs the endonuclease Cas9 to a targeted DNA sequence for site-specific manipulation. In addition to this targeting function, the sgRNA has also been shown to play a role in activating the endonuclease activity of Cas9. This dual function of the sgRNA likely underlies observations that different sgRNAs have varying on-target activities. Currently, our understanding of the relationship between sequence features of sgRNAs and their on-target cleavage efficiencies remains limited, largely due to difficulties in assessing the cleavage capacity of a large number of sgRNAs. In this study, we evaluated the cleavage activities of 218 sgRNAs using *in vitro* Surveyor assays. We found that nucleotides at both PAM-distal and PAM-proximal regions of the sgRNA are significantly correlated with on-target efficiency. Furthermore, we also demonstrated that the genomic context of the targeted DNA, the GC percentage, and the secondary structure of sgRNA are critical factors contributing to cleavage efficiency. In summary, our study reveals important parameters for the design of sgRNAs with high on-target efficiencies, especially in the context of high throughput applications.

The clustered regularly interspaced short palindromic repeat (CRISPR)/CRISPR - associated protein (Cas) system, an adaptive immune system found in many archaea and bacteria, has recently emerged as an efficient and precise tool for genome engineering^1^,^2^,^3^. The system has been further repurposed to regulate gene expression by transcriptional activation or repression^4^,^5^, modify the local chromatin epigenetic status of various loci^6^,^7^, and even target single stranded RNA^8^. Rapid advances in CRISPR-Cas based technology such as these are transforming biological research and hold tremendous potential for future therapeutic applications.

To date, three CRISPR-Cas subtypes have been classified in prokaryotes^9^. Among them, the type II CRISPR-Cas system derived from *Streptococcus pyogenes* is the most commonly used based on its relative simplicity^10^. In particular, the type II CRISPR system utilizes a single endonuclease protein Cas9 to induce DNA cleavage while multiple proteins are required in other subtypes^11^. When coupled with Cas9, two non-coding RNAs: the CRISPR associated RNA (crRNA), required for DNA targeting, and the trans-activating RNA (tracrRNA), necessary for nuclease activity, are sufficient to induce DNA cleavage. These two RNAs can be fused as a chimeric single guide RNA (sgRNA) and further cloned with Cas9 into an expression vector, allowing convenient and efficient delivery of the whole system^12^,^13^.

To direct the Cas9 complex to a desired locus for genetic manipulation, a 20-nucleotide guide sequence found within the sgRNA must be complementary to the target DNA^14^. In addition, a protospacer-adjacent motif (PAM) (3 nucleotides NGG for SpCas9) sequence must be present in the targeted genomic locus. Once bound to the target DNA, two nuclease domains in Cas9, HNH and RuvC, cleave the DNA strands complementary and non-complementary to the guide sequence, leaving a blunt-ended DNA double strand break (DSB)^15^. Thus, in theory, any specific 20 nt genomic sequence followed by a PAM can be targeted. The flexibility of this RNA-guided system enables researchers to perform genome editing for virtually any locus of interest in an easy and quick manner by simply changing the sgRNA in the expression vector.

Given the relatively short length of the guide sequence in the sgRNA (20 nt), targeting specificity has become one major concern in using CRISPR-Cas9, and the off-target effects of the system have been extensively investigated^16^. It has been proposed that the 8–12 PAM-proximal bases, known as the seed sequence, determine targeting specificity by making contacting with the arginine-rich bridge helix (BH) within the recognition (REC) lobe of the Cas9 protein^17^; therefore, selecting sites predicted to have the most specific seed regions with the fewest possible off target mismatches may be crucial to improving on-target efficiency. In contrast, the PAM distal sequence has been suggested to be less important for specificity, and mismatches in this region are more likely to be tolerated.

Despite extensive research on off-target effects, only a limited number of studies have focused on analyzing the on-target cleavage efficiency of the sgRNA/Cas9 complex. It has been observed that the mutagenesis rate of the CRISPR/Cas9 system varies greatly^18^. Further studies have implicated that on-target efficiency of site-directed mutation is highly dependent on the sgRNA given that sgRNAs targeting the same genomic locus show different activities^19^. Moreover, several recent studies have attempted to identify sgRNA sequence determinants that may underlie sgRNA cleavage activity^20^,^21^,^22^,^23^,^24^. Doench *et al.* evaluated the efficiencies of a total of 1,841 sgRNAs in inducing complete loss of a protein and demonstrated that the nucleotide composition at specific positions, especially the one adjacent to the protospacer-adjacent motif (PAM), contributes to the activity of the sgRNA^21^. Based on previous published datasets, Hu *et al.* analyzed the effects of sequence context on sgRNA efficiency and generated models that achieved reasonable predicative power in which the Area Under Curve (AUC) scores were greater than 0.7 in Receiver Operating Characteristic (ROC)^22^. Despite such advances, a large fraction of inefficient sgRNAs are still not predictable with current models, which emphasizes the need to further optimize the design principle of sgRNAs. In addition, many sequence features that are highly likely to be relevant to sgRNA activity, such as the genomic context of the targeted region and the stability of the secondary structure of the sgRNA, have not yet been explored and incorporated into a statistical model. In this study, we comprehensively analyzed the sequence features of sgRNAs and their effects on cleavage activity based on the Surveyor assay system.

Among the host of *in vitro* systems available to evaluate sgRNA performance, the Surveyor nuclease assay is the most commonly used and reliable method. This assay utilizes an enzyme mismatch cleavage system in which heteroduplex DNA with mismatches and indels are cleaved. However, despite its high reliability, the procedure is tedious and time-consuming: it usually takes 10 days from the design of sgRNA to obtain the final assay results. Additionally, it is difficult to multiplex the procedure since cell culture, transfection, and genomic PCR are required for each individual sgRNA assay. Currently, systematic evaluations of sgRNA on-target efficiency based on Surveyor assay are still limited, especially using mammalian cell lines. In this study, we reported the evaluation of the on-target activity for 218 sgRNAs based on the mouse Neuro2A cell line.

## Results

**Figure 1:**
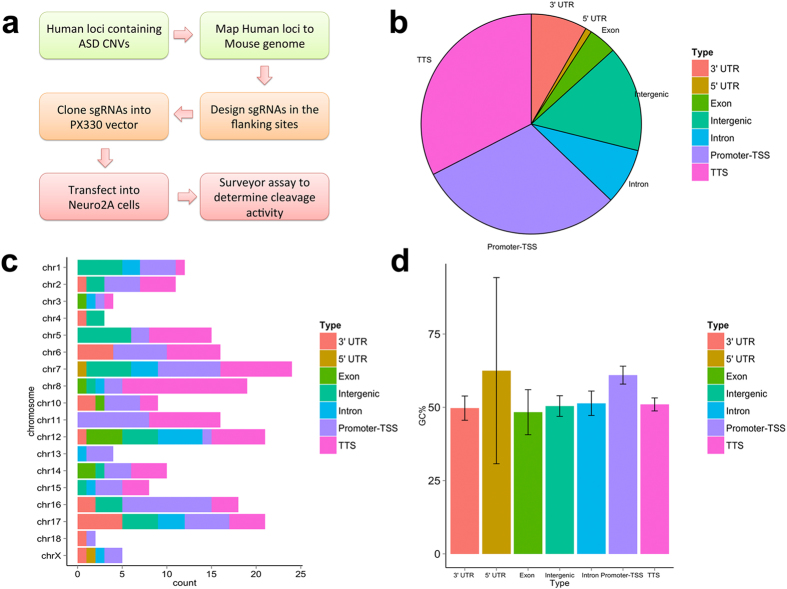
(**a**) Outline of the procedure of the current study. (**b**) Distribution of sgRNAs in various genomic contexts represented by different colors. (**c**) Distribution of sgRNAs across chromosomes. (**d**) The average GC percentages of sgRNAs. The error bar indicated the 95% confidence intervals.

**Figure 2:**
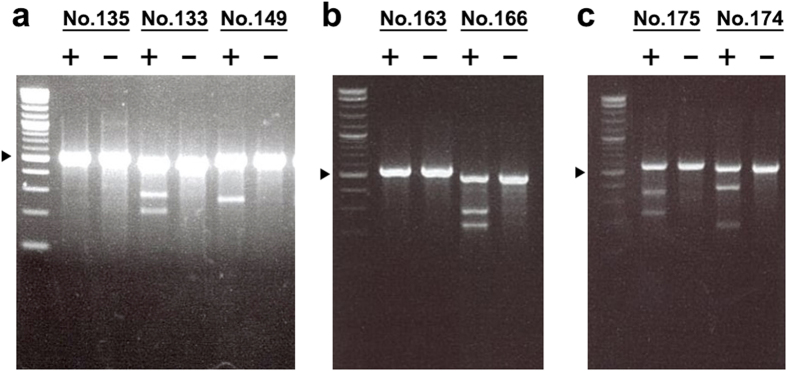
The representative Surveyor assay results of 7 independent sgRNAs. The ID indicates the sgRNA ID shown in the supplementary Table 1. The minus sign indicates the negative control sample that was transfected with pMaxGFP. The plus sign indicates the sample that was transfected with the PX330 vector containing the sgRNA. Among 7 sgRNAs, 5 sgRNAs (No. 133, 149, 166, 174, 175) showed positive cleavage activity and 2 (No. 135, 163) showed no cleavage activity.

We designed and successfully cloned 218 sgRNAs into expression vectors. The experimental design and procedure are briefly outlined in Fig. 1a. The insertion of all guide sequences into the expression vectors was confirmed using Sanger sequencing. Together, these 218 sgRNAs target 153 distinct genomic loci across 18 chromosomes in the mouse genome. The sequences of all sgRNAs as well as detailed annotations including targeted genomic locations, guanine-cytosine (GC) percentages, and genomic contexts are provided in Supplementary Table 1 and illustrated in Fig. 1(b–d). We then performed Surveyor assays using Neuro2A cell line to evaluate the on-target efficiency of these sgRNAs. Representative gel images of the Surveyor assays for seven sgRNA samples are shown in Fig. 2. For each assay, we included one negative control in which a pMax-GFP vector was used for transfection. By comparing PCR bands amplified from the negative controls and sgRNA-transfected samples, we classified the guide sequences in the sgRNAs as Surveyor positive sequences if their cleavage pattern was clearly visible in the sgRNA-transfected samples. Through this analysis, a total of 129 sgRNAs (59%) were determined to be Surveyor positive. Meanwhile, no cleavage was observed for 89 sgRNAs (41%).

### Nucleotide preferences of high efficient sgRNA

**Figure 3:**
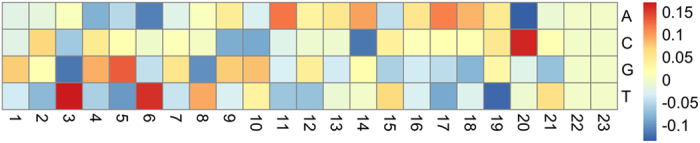
Heatmap plot showing nucleotide frequency change in each position of Surveyor positive sgRNAs compared with that of Surveyor negative sgRNAs. The value of the color scale in each cell of the heatmap indicates the nucleotide frequency difference and is calculated as Frequency^positive^ − Frequency^negative^.

We next set out to explore whether the nucleotide composition of the sgRNAs affected the cleavage results. First, we separated the sgRNA sequences into two groups: Surveyor positive and Surveyor negative. For each group, the occurrences and frequencies of nucleotides (A, C, T, G) at each position were calculated (Table 1). We then compared the nucleotide frequencies in the Surveyor positive sequences with those in the Surveyor negative sequences. A heatmap was subsequently generated to visualize the frequency change between the two groups (Fig. 3). In Surveyor positive sequences, we observed an elevated frequency of thymine (T) at positions 3 and 6, an increased frequency of cytosine (C) and decreased frequency of adenine (A) at position 20, and a host of other nucleotide frequency changes compared to Surveyor negative sequences.

We next asked whether these frequency changes are statistically meaningful or merely represent chance observations. Chi-square analysis was performed for each position of the guide sequence to test if the overall nucleotide composition is different between Surveyor positive and negative sequences. Statistically significant changes were observed at positions 3 and 20 with *P* values of 0.031 and 0.022, respectively (Table 1). Position 3 is located at the PAM-distal region, while position 20 is the base immediately upstream of the PAM sequence. We further calculated the permutation adjusted *P* value for each position based on 10,000 times randomization of the sample labels. The associations of positions 3 and 20 were not significant after corrected by permutation test (permutation *P* value = 0.4762 and 0.371 respectively).

### Evaluation of GC percentage difference

Since the GC percentage of sgRNAs, particularly that of the 6 PAM-proximal bases, has been previously reported to be positively correlated with on-target efficiency^20^,^25^,^26^, we further examined the potential association between GC content and cleavage outcome. We calculated the overall GC percentage for the whole guide sequence, as well as the GC percentages for positions from 1–6, 7–14, and 15–20, in a sliding window manner. Finally, we conducted a Welch two-sample T test, non-parametric Kolmogorov-Smirnov test and logistic regression analysis but did not observe any significant associations (Supplementary Table 2).

### Logistic regression analysis

As a follow-up to the Chi-square test, we tried to evaluate the effect of each nucleotide on cleavage efficiency through logistic regression analysis. The nucleotides in each position were coded as dummy variables, and the nucleotide that showed the lowest frequency change at each given position was set as the baseline level. Similarly, the genomic contexts of the target sequence were also included in the regression analysis where the intergenic region was set as the baseline level. In addition to the sequential features of the sgRNAs, we also evaluated the impacts of several structural features on cleavage efficiency. We assessed the overall secondary structure of each sgRNA measured as single minimum free energy (MFE). Additionally, we analyzed the local secondary structure of the seed region and the effects of the guide sequence on tracrRNA structure. We further speculated that the relationship between GC percentage and cleavage efficiency is likely to be a non-linear one, where sgRNAs with GC percentages that are too high or too low are unfavorable. Thus, we labeled sgRNAs with GC percentages within the range of 40%–60% as “GC normal” and those with GC percentages below 40% or above 60% as “GC abnormal.” These variables were incorporated into the logistic regression model.

**Figure 4:**
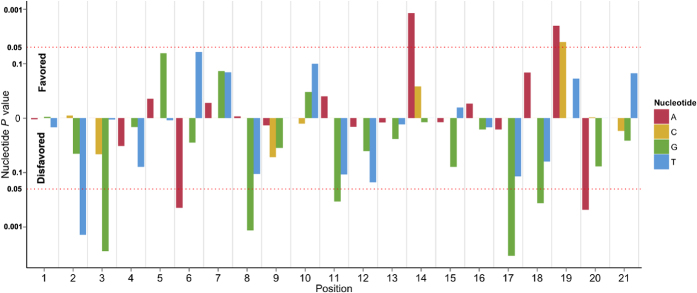
*P*-values of nucleotides from position 1 to 21 assessed by the logistic regression analysis. The y-axis direction indicates whether a given nucleotide is favored or disfavored for cleavage activity.

After logistic regression analysis, a total of 14 variables were found to be significantly correlated with the cleavage results, including the nucleotide present at 10 distinct positions, the condition of being targeted to a promoter-transcription start site (TSS), having a normal range GC, as well as several features of the secondary structures of sgRNA. The position-dependent nucleotide *P* values are illustrated in Fig. 4. The results of significantly associated variables are shown in Table 2, and the complete logistic analysis results are listed in Supplementary Table 3.

To evaluate the performance of the current model, we first tried to examine how well the cleavage activities of sgRNAs used in this study can be predicted by previous methods. We calculated “on-target scores” for our sgRNAs using the standalone Python software proposed in Doench *et al.*’s study^21^. To do so, we updated our sgRNA sequences as the program requires a 30nt sequence including the flanking sequence of the guide sequence. By using the logistic regression, we found a positive correlation between the “on-target score” and the Surveyor cleavage result, though the *P* value is marginal at 0.07. We then assessed the ROC curve of the model fitted based on this score and calculate the area under the ROC curve (AUC) to be 0.57. As a comparison, the AUC score based on our current logistic model is 0.91 and 0.67 when the fitted model is applied to the total training data and from a 20-fold cross-validation, respectively.

## Discussion

In this study, we performed Surveyor assays to evaluate the on-target efficiency of 218 sgRNA sequences. We found that 41% of the sgRNAs showed no cleavage effects based on our assays. To understand what sequence features influence the cleavage outcome, we performed comprehensive statistical analyses that revealed the position-dependent nucleotide preferences associated with positive cleavage results. We further revealed that the genomic contexts of target DNA as well as the GC percentage and secondary structure of sgRNAs also contribute to sgRNA performance. As such, these factors should be considered when designing guide sequences.

Based on Chi-square analysis, we found that position 3 and position 20, a base adjunct to the PAM, are associated with cleavage efficiency. The significant association at position 20 is in line with previous reports^20^,^21^, further supporting the validity of our findings. Studies on the crystal structure of CRISPR/Cas9 reveal that the nucleotide at position 20 induces DNA double strand separation and is responsible for initiating R-loop formation^27^. Using logistic regression analysis, we further revealed that the presence of an adenine at this position has a negative impact on targeting efficiency. Similarly, previous study has observed that possessing an adenine at position 20 resulted in a nearly 50% decrease in the cut rate^26^. Furthermore, other positions of the PAM-proximal seed region were also found as significant variables correlated with on-target efficiency, which supports the importance of the seed region for the proper functioning of the sgRNA/Cas9 complex.

In addition to the PAM-proximal region, we also observed significant correlations between positions in the PAM-distal region and cleavage efficiency. Unlike the proximal region, the PAM-distal region has been considered less important in determining the sgRNA specificity. However, in our study, we show that this region may actually contribute to the on-target efficiencies of sgRNAs. At position 2 and 3, The T and G were found to have a negative effect on cleavage efficiency. Additionally, A at position 6 was identified as significant nucleotide correlated with the cleavage outcome. It has been shown that the backbones of position 2 and 4–6 interact with the REC1 domain of the sgRNA, which is critical for sgRNA:DNA recognition^17^. The nucleotides at these positions might influence this recognition process and thereby affect cleavage performance.

Previous studies have shown that the GC percentage of overall sgRNAs^26^ and the 6 PAM-proximal nucleotides^25^ are positively correlated with efficiency in zebrafish and *Drosophila*, respectively. However, in our study, which is based on a mammalian cell line, we found that sgRNAs with very high or low GC contents are less effective rather than a simple model in which a greater GC percentage always lead to higher activity. In our logistic regression analysis, we demonstrate that sgRNAs with a GC percentage within the range of 40%–60% are favored for efficient on-target cleavage.

Intriguingly, we found that if a target DNA sequence is located at the promoter-TSS region, the sequence has a greater chance to be successfully cleaved compared with a sequence located in the intergenic region. This result is likely related to local chromatin accessibilities in different types of genomic loci. A recent genome-wide Cas9 binding analysis based on Chip-Sequencing demonstrated that chromatin inaccessibility decrease the dCas9 binding and genomic loci commonly accessible in large number of cell types have a significantly higher probability binding to the sgRNA/Cas9 complex^28^,^29^.

Furthermore, our analysis also suggested that the secondary structure of the guide sequence is also an important parameter that should be considered for designing sgRNAs. In particular, the probability of the seed region to form an unfolded structure was identified as the most significant feature. Counterintuitively, our analysis revealed that if the seed sequence is more likely to form secondary structure, the sgRNA has a higher chance of cleaving the target sequence. Loading of the guide RNA into Cas9 has been demonstrated to be a crucial step in converting Cas9 into an active conformation capable of executing its nuclease function^30^. Thus, the secondary structure of the seed region might have a role in facilitating the loading process and may potentially improve the cleavage activity of Cas9.

Recently, two large-scale studies have been reported with the aim to improve the sgRNA design^21^,^24^. Our current study differs from these two studies in several aspects and has unique advantages. In the first study, Doench *et al.* evaluated 1,841 sgRNAs’ efficiency in inducing complete loss of the protein^21^. These sgRNAs were designed to target six cell surface marker genes. By FACS analysis using antibodies specific to these cell surface proteins, the marker-negative cells were isolated and the sequencing was followed to determine highly active sgRNAs in these cells. Given that sgRNAs targeting at intron or UTR regions are unlikely to affect the coding sequence, only sgRNAs targeting the coding sequences (CDS) were analyzed and were used to build the predictive model. However this design has several potential limitations, for example, the sgRNA that induces in-frame mutation is unlikely to be labeled as high-effective despite it may have a high cleavage efficiency; additionally, if the frame-shift mutation induced by the sgRNA occurred downstream of the epitope sites, the sgRNA might show less effects in abolishing the recognition by antibody. In our study, we systemically designed sgRNAs targeting various loci with different genomic contexts across the genome, and most importantly, rather than measuring the effects induced by sgRNA, we directly measured the cleavage efficiency of sgRNAs. In another study, 133 high-activity sgRNAs and 146 low-activity sgRNAs for Cas9_Sp_ together with 82 and 69 sgRNAs for Cas9_St1_ were determined and were used to build the predictive model^24^. Since the support vector machine (SVM) model was adopted in this study, it is difficult to compare the parameters with the current study. Despite differences in the methodology and study design, there was a striking similarity that the most dramatic nucleotide frequency changes were observed at position 20 in all three studies. At this position, either C or G was found with an elevated frequency. The G/C may be preferred to allow RNA/DNA hybridization and might be important for the initiation of the R-loop. Furthermore, in the second study, a strong correlation was observed between the DNase I values of the targeting sites and sgRNA efficiency, supporting the locus accessibility is a critical determinant for the sgRNA activity. Since the DNase I data was not available for the Neuro2A cell, we alternatively retrieved the DNaseI hypersensitivity sites (DHS) of whole mouse brain available at ENCODE project^31^. We merged DHSs from an adult (week 8) and embryonic (day 14.5) mouse and used this collection to represent DHS sites specific to the brain. We then examined how many sgRNA targets overlap with the DHS sites and found that a total of 47 sgRNAs out of 218 overlapped with the brain-specific DHSs. Among them, 32 sgRNAs were located in Promoter/TSS region. Statistical analysis revealed a significant positive correlation between being located at Promoter/TSS and being located in DHS (*P* = 2.7 × 10^−11^). This observation confirms that the promoter/TSS regions have a higher level of chromatin accessibility. This link was further supported by a genome-wide survey of chromatin accessibility of human genome using 125 diverse cell and tissue types, in which it was found that promoters typically exhibit high accessibility across various cell types^32^. In our study we revealed that the secondary structure of the guide sequence of sgRNA is associated with the on-target efficiency and the inclusion of secondary structure variables greatly improves the prediction power of the model. We showed that our logistic regression model performs reasonably well. The detailed parameters of the model are provided and may prove valuable for future studies. The full dataset is also available and can be used as a source for meta-analysis in future studies.

Although our study offers key insights into sgRNA design, attention should be paid for interpreting the results. First, we used cleavage outcome data, which is binary in nature, for our statistical analysis. Although binary responses are easy to understand and interpret, and by this criterion we can clearly separate the sgRNAs into 2 distinct groups, the efficiencies of individual guide sequences might differ within the same group of sgRNAs that showed positive cleavage results. Thus, quantitative outcomes such as cleavage percentage and number of mutations induced by each sgRNA are needed to provide further insight into sgRNA optimization. Secondly, we used 800 ng plasmids for each transfection, which is commonly used for 24-well plate^33^. Based on *in vivo* mutagenesis study of CRISPR/Cas9 in *Drosophila*, the protein level of Cas9 is unlikely to be a critical factor for mutagenesis efficiency, while the amount sgRNA has a more profound impact^25^. Thus, the sgRNA amount may need to be optimized depending on specific experiment condition and cell type.

## Conclusion

Here we report a systematic evaluation of on-target performance of 218 sgRNAs based on *in vitro* Surveyor assay. We found that 41% of sgRNAs in our study showed negative results for cleavage, further emphasizing the need to improve the design of the sgRNA. Through statistical analysis, we found that nucleotide preferences at positions both adjunct and distal to the PAM sequence are significantly correlated with on-target performance. Furthermore, we showed that the genomic contexts of the target region, the optimal GC percentage, and secondary structure of sgRNA are important factors contributing to the cleavage efficiency. Taken together, our study reveals crucial parameters for the design of sgRNAs to achieve high on-target efficiency, particularly in the context of high throughput applications. Future studies are warranted to further replicate our study and improve the state-of-the-art CRISPR/Cas9 technology.

## Methods

### Design and cloning of sgRNA

The sgRNAs were designed to target the flanking sites of various loci that harbor copy number variations (CNVs) associated with autism spectrum disorder (ASD). The top 100 most frequently occurred ASD CNVs were retrieved from the SFARI CNV database^34^. We then used Ensembl Compara API to determine the syntenic regions in the corresponding mouse genome. The sgRNAs were designed at the flanking sites of such mouse loci regardless their genomic contexts. The DNA sequences of selected regions were obtained from the Ensembl database (GRCm38.p3) and were subsequently used as inputs for the CRISPR design tool (http://crispr.mit.edu). Then, candidate sgRNAs with the highest scores (generally indicating fewest potential off-targets) were selected and synthesized. Two complementary oligonucleotides of sgRNAs were annealed, phosphorylated, and cloned into the BbsI sites of pX330 CRISPR/Cas9 vector (Addgene plasmid ID 42230).

### Cell culture and transfection

Neuro2A (N2A) cells were cultured in Dulbecco’s modified Eagle’s Medium (DMEM) supplemented with 10% fetal bovine serum (Life Technology), 100 units penicillin, and 100 μg Streptomycin (Nacalai) and incubated at 37 °C with 5% CO_2_. The cells were seeded into 24-well plates (FALCON) to reach 1 × 10^5^ cells per well. Plasmids (800 ng) were transfected using Lipo3000 reagents. N2A cells were harvested 48 hours post-transfection.

### Surveyor assay

N2A cells transfected with both empty and sgRNA-containing PX330 vectors were treated with buffer containing proteinase K, and genomics DNA was then extracted by ethanol precipitation. Genomic PCR was conducted to amplify a 400–700 bp region containing the sgRNA target. PCR products were gel purified with Wizard SV Gel and the PCR CleanUp kit (Promega). 800 ng of each purified PCR product was mixed and re-annealed to form heteroduplexes, which were subsequently treated with SURVEYOR nuclease and SURVEYOR enhancer S (Transgenomics) following the manufacturer’s recommended protocol. The final product was separated on a 3% TAE Agarose gel and stained with ethidium bromide.

### Statistical Analysis

The R environment (version 3.1.3) was used for statistical analyses^35^. The two-sided *P* value < 0.05 was regarded as the level of statistical significance. Categorical variables were analyzed using Chi-square test. Independent two-sample t-tests and Kolmogorov-Smirnov test were used in the comparison of means between groups. Logistic regression was used to determine factors independently correlated with cleavage efficiency. To adjust for multiple testing, we further calculate permutation *P* values based on 10,000 times randomization. In each cycle of the permutation test, 129 and 89 sgRNAs were randomly assigned as positive and negative sequences, standard Chi-square test was followed and the smallest *P* value among all 21 positions was recorded to construct an empirical frequency distribution of the smallest *P* values. After 10,000 repeats of this procedure, the permutated *P* value is determined by comparing the original *P* value from the real data with the empirical *P* value distribution. We used annotatePeaks.pl program from the Homer Chip-Seq software to annotate the genomic context of each sgRNA target^36^ based on the following categories: 3′ UTR, Promoter-TSS, TTS (Transcription termination site), 5′ UTR, intron, exon and intergenic region. To evaluate the performance of the logistic regression model, we performed Receiver operating characteristics (ROC) analysis in two settings. In the first setting, we trained the model using all samples and then examined how well the model can predict the cleavage results of the input samples. To prevent over-fitting, in the second setting, we repeated the modeling based on a 20-fold cross-validation (CV) and calculated the mean AUC value from the 20 times iteration.

### Secondary structure analysis of sgRNAs

The MFE of each sgRNA was predicted using RNAfold with the default parameters^37^. RNAplfold can compute local pair probabilities and has been used to model RNA co-transcriptional folding by estimating the relative stabilities of all local structures based on a sliding window approach^38^. As such, we used RNAplfold to assess the probability that the entire seed sequence is unpaired (i.e. no folding structure) by scanning the seed region using a sliding window and averaging the probability over all windows which contain the seed region. We set the window size W = 21 which is the length of the guide sequence appended with an additional G used for U6 promoter (GN_20_), and U = 12 which is the length of the seed sequence. Finally, we also estimated the effect of the guide sequence on the tracrRNA structure using the dot plot of the base-pairing matrix predicted by RNAfold. In brief, for each nucleotide on the tracrRNA, we calculated its maximum and average base pairing probability with nucleotides on the guide sequence from the base-pairing matrix. We then averaged each individual probability over all nucleotides on tracrRNA and calculated the overall probability that the tracrRNA structure interacts with the guide sequences.

## Additional Information

**How to cite this article**: Liu, X. *et al.* Sequence features associated with the cleavage efficiency of CRISPR/Cas9 system. *Sci. Rep.* **6**, 19675; doi: 10.1038/srep19675 (2016).

## Supplementary Material

Supplementary Information

## Figures and Tables

**Table 1:** The occurrences and frequencies of nucleotides at each position in Surveyor positive and negative sequences.

Position	Surveyor Positive Sequences				Surveyor Negative Sequences					
	A	C	G	T	A	C	G	T	*P*value	*P*value (perm)
**1**	35 (27.1%)	25 (19.4%)	43 (33.3%)	26 (20.2%)	26 (29.2%)	19 (21.3%)	23 (25.8%)	21 (23.6%)	0.694	1
**2**	33 (25.6%)	32 (24.8%)	33 (25.6%)	31 (24%)	24 (27%)	16 (18%)	21 (23.6%)	28 (31.5%)	0.51	1
**3**	33 (25.6%)	21 (16.3%)	24 (18.6%)	51 (39.5%)	22 (24.7%)	20 (22.5%)	27 (30.3%)	20 (22.5%)	0.031*	0.4762
**4**	20 (15.5%)	41 (31.8%)	45 (34.9%)	23 (17.8%)	21 (23.6%)	24 (27%)	23 (25.8%)	21 (23.6%)	0.213	0.9931
**5**	28 (21.7%)	33 (25.6%)	43 (33.3%)	25 (19.4%)	24 (27%)	21 (23.6%)	18 (20.2%)	26 (29.2%)	0.107	0.9048
**6**	32 (24.8%)	26 (20.2%)	29 (22.5%)	42 (32.6%)	32 (36%)	18 (20.2%)	24 (27%)	15 (16.9%)	0.054	0.6909
**7**	26 (20.2%)	32 (24.8%)	36 (27.9%)	35 (27.1%)	20 (22.5%)	21 (23.6%)	20 (22.5%)	28 (31.5%)	0.774	1
**8**	33 (25.6%)	28 (21.7%)	33 (25.6%)	35 (27.1%)	22 (24.7%)	19 (21.3%)	32 (36%)	16 (18%)	0.283	0.9991
**9**	33 (25.6%)	34 (26.4%)	37 (28.7%)	25 (19.4%)	19 (21.3%)	31 (34.8%)	19 (21.3%)	20 (22.5%)	0.39	0.9999
**10**	25 (19.4%)	28 (21.7%)	41 (31.8%)	35 (27.1%)	20 (22.5%)	27 (30.3%)	21 (23.6%)	21 (23.6%)	0.348	0.9996
**11**	33 (25.6%)	26 (20.2%)	35 (27.1%)	35 (27.1%)	12 (13.5%)	20 (22.5%)	27 (30.3%)	30 (33.7%)	0.185	0.9869
**12**	29 (22.5%)	34 (26.4%)	40 (31%)	26 (20.2%)	17 (19.1%)	24 (27%)	24 (27%)	24 (27%)	0.648	1
**13**	28 (21.7%)	37 (28.7%)	33 (25.6%)	31 (24%)	15 (16.9%)	26 (29.2%)	26 (29.2%)	22 (24.7%)	0.825	1
**14**	31 (24%)	37 (28.7%)	39 (30.2%)	22 (17.1%)	13 (14.6%)	36 (40.4%)	25 (28.1%)	15 (16.9%)	0.205	0.9923
**15**	16 (12.4%)	44 (34.1%)	39 (30.2%)	30 (23.3%)	15 (16.9%)	27 (30.3%)	32 (36%)	15 (16.9%)	0.468	1
**16**	30 (23.3%)	41 (31.8%)	30 (23.3%)	28 (21.7%)	16 (18%)	27 (30.3%)	24 (27%)	22 (24.7%)	0.745	1
**17**	38 (29.5%)	33 (25.6%)	33 (25.6%)	25 (19.4%)	16 (18%)	21 (23.6%)	27 (30.3%)	25 (28.1%)	0.167	0.9803
**18**	36 (27.9%)	37 (28.7%)	29 (22.5%)	27 (20.9%)	17 (19.1%)	24 (27%)	27 (30.3%)	21 (23.6%)	0.366	0.9997
**19**	38 (29.5%)	34 (26.4%)	40 (31%)	17 (13.2%)	22 (24.7%)	19 (21.3%)	25 (28.1%)	23 (25.8%)	0.126	0.9394
**20**	13 (10.1%)	63 (48.8%)	26 (20.2%)	27 (20.9%)	21 (23.6%)	29 (32.6%)	20 (22.5%)	19 (21.3%)	0.022*	0.371
**21**	44 (34.1%)	8 (6.2%)	20 (15.5%)	57 (44.2%)	31 (34.8%)	4 (4.5%)	20 (22.5%)	34 (38.2%)	0.545	1

*P* value (perm): *P* value obtained based on 10,000 times permutation test.

**Table 2:** Variables significant associated with on-target efficiency of sgRNA.

	Estimate	Std. Error	Z	*P* value
Unpairing probability of guide sequence	−9.054	2.505	−3.614	0.0003
GC normal	3.143	0.950	3.311	0.0009
Pos_2_T	−2.419	0.900	−2.688	0.0072
Pos_3_G	−2.464	0.847	−2.911	0.0036
Pos_8_G	−2.110	0.804	−2.625	0.0087
Pos_17_G	−2.419	0.814	−2.970	0.0030
Context: promoter TSS	3.862	1.256	3.075	0.0021
Pos_6_A	−2.049	0.898	−2.281	0.0225
Pos_11_G	−1.861	0.854	−2.178	0.0294
Pos_14_A	2.535	1.007	2.518	0.0118
Pos_18_G	−1.798	0.816	−2.205	0.0275
Pos_19_A	1.785	0.768	2.326	0.0200
Pos_19_C	1.827	0.891	2.051	0.0403
Pos_20_A	−2.216	0.959	−2.311	0.0208

TSS: transcription start site; Estimate: estimated effect doses. Std.Error: Standard error; Pos is abbreviated for position.
