# Supplementary material for: Sequence features associated with the cleavage efficiency of CRISPR/Cas9 system
Source: Sci Rep. 2016 Jan 27;6:19675. doi: 10.1038/srep19675 (PMC4728555; doi:10.1038/srep19675)
Supplement: Supplementary Information [file srep19675-s1.pdf]

Title

Sequence features associated with the cleavage efficiency of CRISPR/Cas9 system

Authors

Xiaoxi Liu[a], Ayaka Homma[a], Jamasb Sayadi[b], Shu Yang[c], Jun Ohashi[d], Toru Takumi[a,e]*

Affiliations of all authors

[a] RIKEN Brain Science Institute, Wako, Saitama, Japan

[b] Harvard College, Cambridge, Massachusetts 02138, United States

[c] Department of Computer Science, University of British Columbia, Vancouver, Canada

[d] Department of Biological Sciences, Graduate School of Science, University of Tokyo, Bunkyo, Tokyo, Japan

[e] JST, CREST

*Corresponding author: Toru Takumi, M.D., Ph.D.

RIKEN Brain Science Institute, 2-1 Hirosawa, Wako, Saitama 351-0198, Japan,

E-mail: toru.takumi@riken.jp

Supplementary Table 1: Detailed annotations of the sgRNA guidance sequences

ID	SeqID	Sequence_With_PAM	Chr	Start	End	Strand	GC	Annotation	Distance_to_TSS	Nearest_Refseq	GeneName	GeneType	Guide_Sequence	NormalGC	unpair probability	ave_tracer_impact	max_tracer_impact	SgRNA	Structure	MFE
1	Top_00_3_1	TATCCTACTAACTTTCGACCAGG	chrX	68716557	68716579	minus	40	3' UTR	-38027	NM_008031	Fmr1	protein-coding	UAUCCUACUAACUUUCGACC	YES	0.7233603	1.03E-01	0.815096441	GUAUCCUACUAACUUUCGACCGUUUUAGAGCUAGAAAUAGCAAGUUAAAAUAAGGCUAGUCCGUUAUCAACUUGAAAAAGUGGCACCGAGUCGGUGCUUU	.......((((.((((.....((((((.((((....))))...)))))))))).))))..........((((....))))((((((...))))))..	-20.2
2	TOP_00_5_1	TCACCAAGTGCGCGCGCCAAAGG	chrX	68678389	68678411	plus	65	promoter-TSS	-1	NR_033481	Gm10474	ncRNA	UCACCAAGUGCGCGCGCCAA	NO	0.1517316	1.63E-01	0.998594094	GUCACCAAGUGCGCGCGCCAAGUUUUAGAGCUAGAAAUAGCAAGUUAAAAUAAGGCUAGUCCGUUAUCAACUUGAAAAAGUGGCACCGAGUCGGUGCUUU	.....((((((((.(((((..((((((.((((....))))...)))))))..))).)).)).))).....))))).......((((((...))))))..	-27.8
3	TOP_01_3_1	AGTGGGAGAGTCACGGACTTAGG	chr7	127133216	127133238	plus	55	TTS	4596	NM_009259	Spn	protein-coding	AGUGGGAGAGUCACGGACUU	YES	0.01347702	0.120384736	0.997969974	GAGUGGGAGAGUCACGGACUUGUUUUAGAGCUAGAAAUAGCAAGUUAAAAUAAGGCUAGUCCGUUAUCAACUUGAAAAAGUGGCACCGAGUCGGUGCUUU	........((...((((((.((((((..((((....)))).........)))))).))))))..)).((((....))))((((((...))))))..	-29.1
4	TOP_01_5_1	ATACCGGGGTGTGCGAGGACTGG	chr7	126704017	126704039	minus	65	5' UTR	-192	NM_009898	Coro1a	protein-coding	AUACCGGGGUGUGCGAGGAC	NO	0.07598211	1.49E-01	0.999935275	GAUACCGGGGUGUGCGAGGACGUUUUAGAGCUAGAAAUAGCAAGUUAAAAUAAGGCUAGUCCGUUAUCAACUUGAAAAAGUGGCACCGAGUCGGUGCUUU	((((.((((.((.((......((((((.((((....))))...)))))))...)))).)))).)))).((((....))))((((((...))))))..	-29.2
5	TOP_02_3_1	TGAGTAAATTATCACCCGGAAGG	chr7	56020606	56020628	minus	40	Intergenic	1044	NM_153578	Nipa1	protein-coding	UGAGUAAAUUAUCACCCGGA	YES	0.5771943	4.82E-02	0.934670137	GUGAGUAAAUUAUCACCCGGAGUUUUAGAGCUAGAAAUAGCAAGUUAAAAUAAGGCUAGUCCGUUAUCAACUUGAAAAAGUGGCACCGAGUCGGUGCUUU	((((........)))).((((((((((.((((....))))...)))))))........))))......((((....))))((((((...))))))..	-23.8
6	TOP_02_3_2	TCCAAATTATAGCCGGATGTTGG	chr7	55977688	55977710	minus	40	TTS	-15168	NM_001033396	A230056P14Rik	ncRNA	UCCAAAUUAUAGCCGGAUGU	YES	0.3796269	1.22E-01	0.985865171	GUCCAAAUUAUAGCCGGAUGUGUUUUAGAGCUAGAAAUAGCAAGUUAAAAUAAGGCUAGUCCGUUAUCAACUUGAAAAAGUGGCACCGAGUCGGUGCUUU	.........(((((.((((..((((((..((((....)))).........))))))..)))))))))..((((....))))((((((...))))))..	-26
7	TOP_02_5_2	ATTGCCGCGGCGTACCTATCAGG	chr7	55794174	55794196	minus	60	promoter-TSS	-37	NM_146190	Tubgcp5	protein-coding	AUUGCCGCGGCGUACCUAUC	YES	0.04079466	0.135952475	0.99813422	GAUUGCCGCGGCGUACCUAUCGUUUUAGAGCUAGAAAUAGCAAGUUAAAAUAAGGCUAGUCCGUUAUCAACUUGAAAAAGUGGCACCGAGUCGGUGCUUU	.......((((..(((((...((((((.((((....))))...))))))).))).)).)).))))......((((....))))((((((...))))))..	-26.7
8	TOP_03_3_1	ATCCCTAAGAAGTCAACGGAAGG	chr17	90034107	90034129	minus	45	3' UTR	-654877	NR_045952	Gm4719	ncRNA	AUCCCUAAGAAGUCAACGGA	YES	0.2305736	1.00E-01	0.997271502	GAUCCCUAAGAAGUCAACGGAGUUUUAGAGCUAGAAAUAGCAAGUUAAAAUAAGGCUAGUCCGUUAUCAACUUGAAAAAGUGGCACCGAGUCGGUGCUUU	...............((((((((((((.((((....))))...)))))))........))))))....((((....))))((((((...))))))..	-23
9	TOP_03_5_1	GATCCTGGCCCAGTTACAACAGG	chr17	91092501	91092523	plus	55	intron	290	NM_020252	Nrxn1	protein-coding	GAUCCUGGCCCAGUUACAAC	YES	0.5436166	1.47E-01	0.999901105	GGAUCCUGGCCCAGUUACAACGUUUUAGAGCUAGAAAUAGCAAGUUAAAAUAAGGCUAGUCCGUUAUCAACUUGAAAAAGUGGCACCGAGUCGGUGCUUU	(((..((((((..........((((((.((((....))))...))))))))..)))))))))).......((((....))))((((((...))))))..	-26.8
10	TOP_03_5_2	AGGAGCTCAATTTGGGCGCGGGG	chr17	91092572	91092594	minus	60	intron	-219	NM_020252	Nrxn1	protein-coding	AGGAGCUCAAUUUGGGCGCG	YES	0.159729	7.14E-02	0.850457808	GAGGAGCUCAAUUUGGGCGCGGUUUUAGAGCUAGAAAUAGCAAGUUAAAAUAAGGCUAGUCCGUUAUCAACUUGAAAAAGUGGCACCGAGUCGGUGCUUU	(((...)))......((((..((((((..((((....)))).........))))))).))))........((((....))))((((((...))))))..	-24.5
11	TOP_04_3_1	GTCCGGTGGTGAGTCCCACGTGG	chr3	97673703	97673725	sss	70	TTS	-15502	NM_182997	Prkab2	protein-coding	GUCCGGUGGUGAGUCCCACG	NO	0.01241089	6.83E-02	0.510595425	GGUCCGGUGGUGAGUCCCACGGUUUUAGAGCUAGAAAUAGCAAGUUAAAAUAAGGCUAGUCCGUUAUCAACUUGAAAAAGUGGCACCGAGUCGGUGCUUU	(((.(((.....((((.....((((((.((((....))))...))))))))..))))...)))..))).((((....))))((((((...))))))..	-24
12	TOP_04_5_1	ATTCACTTGTGCCACCGTATGGG	chr3	96867871	96867893	plus	45	intron	35208	NM_021517	Pdzk1	protein-coding	AUUCACUUGUGCCACCGUAU	YES	0.1696466	1.55E-01	0.747928026	GAUUCACUUGUGCCACCGUAUGUUUUAGAGCUAGAAAUAGCAAGUUAAAAUAAGGCUAGUCCGUUAUCAACUUGAAAAAGUGGCACCGAGUCGGUGCUUU	(((.........(((......((((((.((((....))))...)))))))).)))..........))).((((....))))((((((...))))))..	-21.1
13	TOP_04_5_2	AATGCCATTCGGGGTCAGCCTGG	chr3	96868494	96868516	plus	60	exon	35831	NM_021517	Pdzk1	protein-coding	AAUGCCAUUCGGGGUCAGCC	YES	0.003767217	1.17E-01	0.999891355	GAAUGCCAUUCGGGGUCAGCCGUUUUAGAGCUAGAAAUAGCAAGUUAAAAUAAGGCUAGUCCGUUAUCAACUUGAAAAAGUGGCACCGAGUCGGUGCUUU	..........((((...((((((((((.((((....))))...)))))))..)))).))))......((((....))))((((((...))))))..	-27.3
14	TOP_05_3_1	ACGCATACCCCTAGGTGGTGAGG	chr5	135367786	135367808	minus	60	TTS	2156	NM_145414	Nsun5	protein-coding	ACGCAUACCCCUAGGUGGUG	YES	0.004575406	2.38E-02	0.206909402	GACGCAUACCCCUAGGUGGUGGUUUUAGAGCUAGAAAUAGCAAGUUAAAAUAAGGCUAGUCCGUUAUCAACUUGAAAAAGUGGCACCGAGUCGGUGCUUU	..(((.((((....)))))))((((((.((((....))))...)))))))..................((((....))))((((((...))))))..	-23.5
15	TOP_05_5_1	GCGCTTCGCCTCGGCCTAGAGGG	chr5	134314931	134314953	minus	70	promoter-TSS	182	NM_010365	Gtf2i	protein-coding	GCGCUUCGCCUCGGCCUAGA	NO	0.00588198	7.10E-02	0.988922193	GGCGCUUCGCCUCGGCCUAGAGUUUUAGAGCUAGAAAUAGCAAGUUAAAAUAAGGCUAGUCCGUUAUCAACUUGAAAAAGUGGCACCGAGUCGGUGCUUU	((((...))))..(((((...((((((.((((....))))...))))))).)))))............((((....))))((((((...))))))..	-29.6
16	TOP_05_5_2	ATACGTGGTTTATGTACTACAGG	chr5	134316619	134316641	plus	35	Intergenic	-1870	NM_010365	Gtf2i	protein-coding	AUACGUGGUUUAUGUACUAC	NO	0.1465534	4.11E-02	0.943162073	GAUACGUGGUUUAUGUACUACGUUUUAGAGCUAGAAAUAGCAAGUUAAAAUAAGGCUAGUCCGUUAUCAACUUGAAAAAGUGGCACCGAGUCGGUGCUUU	...(((((((.......)))))))......((((....))))(((((...((((((.....)).)))).)))))........((((((...))))))..	-24
17	TOP_06_3_1	AGAATCTCTTGTGCATCGTGTGG	chr16	32777182	32777204	plus	45	TTS	20242	NM_146071	Muc20	protein-coding	AGAAUCUCUUGUGCAUCGUG	YES	0.563589	7.41E-02	0.284963762	GAGAAUCUCUUGUGCAUCGUGGUUUUAGAGCUAGAAAUAGCAAGUUAAAAUAAGGCUAGUCCGUUAUCAACUUGAAAAAGUGGCACCGAGUCGGUGCUUU	......((((...((.......))...))))(((....)))((((((...((((((.....)).)))).))))))........((((((...))))))..	-19.1
18	TOP_06_5_1	ACACTCCGGTGCGTGCGTTAGGG	chr16	31428781	31428803	minus	60	promoter-TSS	-39	NM_175177	Bdh1	protein-coding	ACACUCCGGUGCGUGCGUUA	YES	0.2390251	1.25E-01	0.99575976	GACACUCCGGUGCGUGCGUUAGUUUUAGAGCUAGAAAUAGCAAGUUAAAAUAAGGCUAGUCCGUUAUCAACUUGAAAAAGUGGCACCGAGUCGGUGCUUU	.......(((.((..((.(((.((((((.((((....))))...))))))))).))..)))))......((((....))))((((((...))))))..	-27.5
19	TOP_07_3_1	GTTCGTTCAACATAGCGTTCTGG	chr16	18973275	18973297	plus	45	Intergenic	96536	NM_010435	Hira	protein-coding	GUUCGUUCAACAUAGCGUUC	YES	0.2548917	1.23E-01	0.997100516	GGUUCGUUCAACAUAGCGUUCGUUUUAGAGCUAGAAAUAGCAAGUUAAAAUAAGGCUAGUCCGUUAUCAACUUGAAAAAGUGGCACCGAGUCGGUGCUUU	(((.((.....((.((((.((.((((((.((((....))))...)))))))))).)))))).))..))).((((....))))((((((...))))))..	-24.3
20	TOP_07_3_2	TGTCTACCTTGGGGTCAAGCTGG	chr16	18969849	18969871	minus	55	3' UTR	-93110	NM_010435	Hira	protein-coding	UGUCUACCUUGGGGUCAAGC	YES	0.05639345	8.69E-02	0.964831298	GUGUCUACCUUGGGGUCAAGCGUUUUAGAGCUAGAAAUAGCAAGUUAAAAUAAGGCUAGUCCGUUAUCAACUUGAAAAAGUGGCACCGAGUCGGUGCUUU	.............((...((((((((.((((....))))...)))))))...)))...))........((((....))))((((((...))))))..	-21.8
21	TOP_07_5_1	GACGCCCACCCGACAGTTCCTGG	chr16	17562117	17562139	minus	70	promoter-TSS	-243	NM_011028	P2rx6	protein-coding	GACGCCCACCCGACAGUUCC	NO	0.3994435	9.87E-02	0.996087457	GGACGCCCACCCGACAGUUCCGUUUUAGAGCUAGAAAUAGCAAGUUAAAAUAAGGCUAGUCCGUUAUCAACUUGAAAAAGUGGCACCGAGUCGGUGCUUU	((.........))(((((((..((((((.((((....))))...)))))))..)))).)))........((((....))))((((((...))))))..	-24.5
22	TOP_07_5_2	GCTAACCAGGAACTGTCGGGTGG	chr16	17562112	17562134	plus	60	promoter-TSS	238	NM_011028	P2rx6	protein-coding	GCUAACCAGGAACUGUCGGG	YES	0.01901301	1.40E-01	0.99438964	GGCUAACCAGGAACUGUCGGGGUUUUAGAGCUAGAAAUAGCAAGUUAAAAUAAGGCUAGUCCGUUAUCAACUUGAAAAAGUGGCACCGAGUCGGUGCUUU	((.((((..(((.(((((...((((((.((((....))))...)))))))..))).)))))))))))).((((....))))((((((...))))))..	-26.4
23	TOP_08_3_1	GAACCAGGGATATTAGCCCTGGG	chrX	75576145	75576167	plus	50	intron	2075	NM_175122	Rab39b	protein-coding	GAACCAGGGAUAUUAGCCCU	YES	0.09185586	1.44E-01	0.999970943	GGAACCAGGGAUAUUAGCCCUGUUUUAGAGCUAGAAAUAGCAAGUUAAAAUAAGGCUAGUCCGUUAUCAACUUGAAAAAGUGGCACCGAGUCGGUGCUUU	........((((..(((((.((((((((.((((....))))...)))))))).)))))))))........((((....))))((((((...))))))..	-32
24	TOP_08_3_2	ATCGGCCGCGAGAACATCACTGG	chrX	75578100	75578122	plus	60	5' UTR	120	NM_175122	Rab39b	protein-coding	AUCGGCCGCGAGAACAUCAC	YES	0.03091626	0.110000312	0.9721456	GAUCGGCCGCGAGAACAUCACGUUUUAGAGCUAGAAAUAGCAAGUUAAAAUAAGGCUAGUCCGUUAUCAACUUGAAAAAGUGGCACCGAGUCGGUGCUUU	(((.((((...(((((.....)))))...((((....))))...........)))).)))..........((((....))))((((((...))))))..	-23.4
25	TOP_08_5_1	GAGGGGTACGATGCTAATCCTGG	chrX	73227952	73227974	plus	55	promoter-TSS	-343	NM_007978	F8a	protein-coding	GAGGGGUACGAUGCUAAUCC	YES	0.04099837	1.22E-01	0.806829153	GGAGGGGUACGAUGCUAAUCCGUUUUAGAGCUAGAAAUAGCAAGUUAAAAUAAGGCUAGUCCGUUAUCAACUUGAAAAAGUGGCACCGAGUCGGUGCUUU	.....((((((..((((..((((((((.((((....))))...)))))))..)).)))).)).)))).((((....))))((((((...))))))..	-23.5
26	TOP_09_3_1	AGGCTTGATGCCCCCAGTATAGG	chr7	63607845	63607867	plus	55	intron	163084	NM_130880	Otud7a	protein-coding	AGGCUUGAUGCCCCCAGUAU	YES	0.2059139	8.01E-02	0.939123569	GAGGCUUGAUGCCCCCAGUAUGUUUUAGAGCUAGAAAUAGCAAGUUAAAAUAAGGCUAGUCCGUUAUCAACUUGAAAAAGUGGCACCGAGUCGGUGCUUU	..(((.....)))...(((.((((((((.((((....))))...))))))))..)))...........((((....))))((((((...))))))..	-24.6
27	TOP_09_3_2	GTGGCAGTTGTCCATCGAACTGG	chr7	63618618	63618640	plus	55	intron	173857	NM_130880	Otud7a	protein-coding	GUGGCAGUUGUCCAUCGAAC	YES	0.2162574	2.17E-02	0.257289657	GGUGGCAGUUGUCCAUCGAACGUUUUAGAGCUAGAAAUAGCAAGUUAAAAUAAGGCUAGUCCGUUAUCAACUUGAAAAAGUGGCACCGAGUCGGUGCUUU	(((((.......)))))....((((((.((((....))))...)))))))..................((((....))))((((((...))))))..	-22
28	TOP_09_5_1	ATCCACTGACCAGTTCGTGGAGG	chr7	63096735	63096757	plus	55	Intergenic	115780	NM_007390	Chrna7	protein-coding	AUCCACUGACCAGUUCGUGG	YES	0.003955997	8.43E-02	0.478190048	GAUCCACUGACCAGUUCGUGGGUUUUAGAGCUAGAAAUAGCAAGUUAAAAUAAGGCUAGUCCGUUAUCAACUUGAAAAAGUGGCACCGAGUCGGUGCUUU	(((..((.(((.((((.....((((((.((((....))))...))))))))..)))).))).)).))).((((....))))((((((...))))))..	-24.4
29	TOP_09_5_2	TTTAGATACGTGCATTGCGATGG	chr7	63096759	63096781	minus	40	Intergenic	-115756	NM_007390	Chrna7	protein-coding	UUUAGAUACGUGCAUUGCGA	YES	0.05564989	1.29E-01	0.979902032	GUUUAGAUACGUGCAUUGCGAGUUUUAGAGCUAGAAAUAGCAAGUUAAAAUAAGGCUAGUCCGUUAUCAACUUGAAAAAGUGGCACCGAGUCGGUGCUUU	.....((((((.((...((..((((((.((((....))))...)))))))...))..)).)).)))).((((....))))((((((...))))))..	-24.1
30	TOP_09_5_3	GGTAGACCTTTAGACCATTATGG	chr8	63097001	63097023	plus	40	intron	145667	NM_023689	Spock3	protein-coding	GGUAGACCUUUAGACCAUUA	YES	0.4314003	1.45E-01	0.994721952	GGGUAGACCUUUAGACCAUUAGUUUUAGAGCUAGAAAUAGCAAGUUAAAAUAAGGCUAGUCCGUUAUCAACUUGAAAAAGUGGCACCGAGUCGGUGCUUU	((((((.((((..........((((((.((((....))))...)))))))))))))).)))........((((....))))((((((...))))))..	-24.7
31	TOP_10_5_1	GCCCAAGTCTCTCGCTCCGTTGG	chr11	83850718	83850740	plus	65	promoter-TSS	-140	NM_009330	Hnf1b	protein-coding	GCCCAAGUCUCUCGCUCCGU	NO	0.3574325	9.87E-02	0.602851652	GGCCCAAGUCUCUCGCUCCGUGUUUUAGAGCUAGAAAUAGCAAGUUAAAAUAAGGCUAGUCCGUUAUCAACUUGAAAAAGUGGCACCGAGUCGGUGCUUU	(((....)))(((.((((.........)))).))).....((((((...((((((.....)).)))).))))))........((((((...))))))..	-22.5
32	TOP_12_3_1	AAAGGGCGGTGCCGGCACGCAGG	chr6	106800085	106800107	plus	75	promoter-TSS	-9	NM_021449	Crbn	protein-coding	AAAGGGCGGUGCCGGCACGC	NO	0.007603928	1.42E-01	0.998564716	GAAAGGGCGGUGCCGGCACGCGUUUUAGAGCUAGAAAUAGCAAGUUAAAAUAAGGCUAGUCCGUUAUCAACUUGAAAAAGUGGCACCGAGUCGGUGCUUU	.....(((((.((.(((....((((((.((((....))))...)))))))...))).)))))))....((((....))))((((((...))))))..	-28.4
33	TOP_12_5_1	TCTCAGTGGCAAGGCGAACCCGG	chr6	103510650	103510672	plus	60	promoter-TSS	-215	NM_007697	Chl1	protein-coding	UCUCAGUGGCAAGGCGAACC	YES	0.05715874	9.85E-02	0.81384429	GUCUCAGUGGCAAGGCGAACCGUUUUAGAGCUAGAAAUAGCAAGUUAAAAUAAGGCUAGUCCGUUAUCAACUUGAAAAAGUGGCACCGAGUCGGUGCUUU	......(((((..(((...((((((((.((((....))))...)))))))..))...))).))))).((((....))))((((((...))))))..	-23.5
34	TOP_13_3_1	ATCCACCGCTCCACCTGACGAGG	chr11	62895363	62895385	plus	65	TTS	15884	NM_026210	Tvp23b	protein-coding	AUCCACCGCUCCACCUGACG	NO	0.05047072	8.67E-02	0.683290835	GAUCCACCGCUCCACCUGACGGUUUUAGAGCUAGAAAUAGCAAGUUAAAAUAAGGCUAGUCCGUUAUCAACUUGAAAAAGUGGCACCGAGUCGGUGCUUU	........((((.(((....)))....))))..........((((((...((((((.....)).)))).))))))........((((((...))))))..	-21.7
35	TOP_13_3_2	TAAGTACGGCCATCCTCGTCAGG	chr11	62895376	62895398	minus	55	TTS	-15897	NM_026210	Tvp23b	protein-coding	UAAGUACGGCCAUCCUCGUC	YES	0.03823941	1.23E-01	0.953026756	GUAAGUACGGCCAUCCUCGUCGUUUUAGAGCUAGAAAUAGCAAGUUAAAAUAAGGCUAGUCCGUUAUCAACUUGAAAAAGUGGCACCGAGUCGGUGCUUU	......((((....(((....((((((.((((....))))...))))))).)))......))))......((((....))))((((((...))))))..	-22.3
36	TOP_13_3_3	AACTTAAGGGGGGCAAGCGCTGG	chr11	62895537	62895559	minus	60	TTS	-16058	NM_026210	Tvp23b	protein-coding	AACUUAAGGGGGGCAAGCGC	YES	0.02380322	1.14E-01	0.999728113	GAACUUAAGGGGGGCAAGCGCGUUUUAGAGCUAGAAAUAGCAAGUUAAAAUAAGGCUAGUCCGUUAUCAACUUGAAAAAGUGGCACCGAGUCGGUGCUUU	........((.((((.(((..((((((.((((....))))...)))))))...))).)))).))....((((....))))((((((...))))))..	-28.6
37	TOP_13_3_4	GTCCTAGGGTGGTACCCAAGAGG	chr11	62895695	62895717	minus	60	TTS	-16216	NM_026210	Tvp23b	protein-coding	GUCCUAGGGUGGUACCCAAG	YES	0.02199683	0.024361764	0.784326868	GGUCCUAGGGUGGUACCCAAGGUUUUAGAGCUAGAAAUAGCAAGUUAAAAUAAGGCUAGUCCGUUAUCAACUUGAAAAAGUGGCACCGAGUCGGUGCUUU	...(((.((((...)))).)))........((((....))))(((((...((((((.....)).)))).)))))........((((((...))))))..	-24.2
38	TOP_13_5_1	CCCGTCATCCATTGGTCGACGGG	chr11	59662379	59662401	plus	60	promoter-TSS	-105	NM_012027	Mprip	protein-coding	CCCGUCAUCCAUUGGUCGAC	YES	0.2579091	5.78E-02	0.877869673	GCCCGUCAUCCAUUGGUCGACGUUUUAGAGCUAGAAAUAGCAAGUUAAAAUAAGGCUAGUCCGUUAUCAACUUGAAAAAGUGGCACCGAGUCGGUGCUUU	..........((((((...((((((.((((....))))...)))))))..))))))..........((((....))))((((((...))))))..	-24.2
39	TOP_13_5_2	AGTGCGCGCGGCGCCCAGCTCGG	chr11	59662486	59662508	plus	80	promoter-TSS	2	NM_012027	Mprip	protein-coding	AGUGCGCGCGGCGCCCAGCU	NO	9.47E-05	1.28E-01	0.923428652	GAGUGCGCGCGGCGCCCAGCUGUUUUAGAGCUAGAAAUAGCAAGUUAAAAUAAGGCUAGUCCGUUAUCAACUUGAAAAAGUGGCACCGAGUCGGUGCUUU	((..(((.((...(((....((((((((.((((....))))...)))))))).)))..)).))))..)).((((....))))((((((...))))))..	-27.7
40	TOP_13_5_3	CCAGCTCGGTTCGTTCGCGGTGG	chr11	59662500	59662522	plus	70	promoter-TSS	16	NM_012027	Mprip	protein-coding	CCAGCUCGGUUCGUUCGCGG	NO	0.06587034	1.52E-01	0.838172	GCCAGCUCGGUUCGUUCGCGGGUUUUAGAGCUAGAAAUAGCAAGUUAAAAUAAGGCUAGUCCGUUAUCAACUUGAAAAAGUGGCACCGAGUCGGUGCUUU	(((.(((((((((....)))(((((((.((((....))))...)))))))...((((.....(((.......))).....)))))))))))).)))....	-23.8
41	TOP_14_3_1	AGTAGAACAGATGCGCATCCTGG	chr15	89591978	89592000	minus	50	promoter-TSS	66	NM_026817	Rabl2	protein-coding	AGUAGAACAGAUGCGCAUCC	YES	0.1293609	1.33E-01	0.972008598	GAGUAGAACAGAUGCGCAUCCGUUUUAGAGCUAGAAAUAGCAAGUUAAAAUAAGGCUAGUCCGUUAUCAACUUGAAAAAGUGGCACCGAGUCGGUGCUUU	((((.((((.(((..((....((((((.((((....))))...)))))))...))..))).)).)).)).)))).........((((((...))))))..	-23.7
42	TOP_14_5_1	CGTGGACAGATCCGTGATACAGG	chr15	88913738	88913760	minus	55	TTS	-40669	NM_172818	Ttll8	protein-coding	CGUGGACAGAUCCGUGAUAC	YES	0.1382333	0.091082194	0.859821825	GCGUGGACAGAUCCGUGAUACGUUUUAGAGCUAGAAAUAGCAAGUUAAAAUAAGGCUAGUCCGUUAUCAACUUGAAAAAGUGGCACCGAGUCGGUGCUUU	....((((((..((........((((((.((((....))))...)))))))..)))).)))).......((((....))))((((((...))))))..	-22.4
43	TOP_16_3_1	CAGAGTTGTCTGTAGCGGAAAGG	chr1	94052556	94052578	plus	50	promoter-TSS	-14	NM_008798	Pdcd1	protein-coding	CAGAGUUGUCUGUAGCGGAA	YES	0.04957221	1.63E-01	0.999554564	GCAGAGUUGUCUGUAGCGGAAGUUUUAGAGCUAGAAAUAGCAAGUUAAAAUAAGGCUAGUCCGUUAUCAACUUGAAAAAGUGGCACCGAGUCGGUGCUUU	...((((((...(((((((((((((..((((....)))).........)))))))..))))))))))))))........((((((...))))))..	-30.1
44	TOP_16_5_1	GCTGATTAGCGGCCCGCGCCTGG	chr1	91801211	91801233	plus	75	promoter-TSS	-255	NM_007855	Twist2	protein-coding	GCUGAUUAGCGGCCCGCGCC	NO	9.34E-06	1.49E-01	0.941822528	GGCUGAUUAGCGGCCCGCGCCGUUUUAGAGCUAGAAAUAGCAAGUUAAAAUAAGGCUAGUCCGUUAUCAACUUGAAAAAGUGGCACCGAGUCGGUGCUUU	(((.(((((((((....)))((((((.((((....))))...)))))))...))))))).)))....((((....))))((((((...))))))..	-30.1
45	TOP_16_5_2	AACTGAAAGTGCCGCCCGGGGGG	chr1	91801312	91801334	plus	65	promoter-TSS	-154	NM_007855	Twist2	protein-coding	AACUGAAAGUGCCGCCCGGG	NO	0.01240945	1.51E-01	0.858169521	GAACUGAAAGUGCCGCCCGGGGUUUUAGAGCUAGAAAUAGCAAGUUAAAAUAAGGCUAGUCCGUUAUCAACUUGAAAAAGUGGCACCGAGUCGGUGCUUU	..(((((..(((((((....((((((.((((....))))...)))))))..)))..............((((....))))))))....)))))....	-23.6
46	TOP_16_5_3	AGCGAGGGCGGCGCGCTGATTGG	chr1	91801398	91801420	minus	75	promoter-TSS	68	NM_007855	Twist2	protein-coding	AGCGAGGGCGGCGCGCUGAU	NO	0.01022552	1.44E-01	0.996575459	GAGCGAGGGCGGCGCGCUGAUGUUUUAGAGCUAGAAAUAGCAAGUUAAAAUAAGGCUAGUCCGUUAUCAACUUGAAAAAGUGGCACCGAGUCGGUGCUUU	....((.(((((.(((((..((((((((.((((....))))...))))))))).)))..))))))).)).((((....))))((((((...))))))..	-29.2
47	TOP_17_2_3_1	AGCTGTTTGTCACCCGTTGGTGG	chr14	64591262	64591284	plus	55	promoter-TSS	88	NR_037239	Mir3078	ncRNA	AGCUGUUUGUCACCCGUUGG	YES	0.01854121	8.95E-02	0.98416278	GAGCUGUUUGUCACCCGUUGGGUUUUAGAGCUAGAAAUAGCAAGUUAAAAUAAGGCUAGUCCGUUAUCAACUUGAAAAAGUGGCACCGAGUCGGUGCUUU	.((((........((((...)))).....))))........((((((...((((((.....)).)))).))))))........((((((...))))))..	-22.9
48	TOP_17_2_3_2	CTGGAGTGAGCGAAGGCGATCGG	chr14	64591320	64591342	plus	60	TTS	146	NR_037239	Mir3078	ncRNA	CUGGAGUGAGCGAAGGCGAU	YES	0.0468165	1.14E-01	0.994931096	GCUGGAGUGAGCGAAGGCGAUGUUUUAGAGCUAGAAAUAGCAAGUUAAAAUAAGGCUAGUCCGUUAUCAACUUGAAAAAGUGGCACCGAGUCGGUGCUUU	(((......)))...(((..((((((((.((((....))))...))))))))..)))............((((....))))((((((...))))))..	-27
49	TOP_17_2_3_3	GCGATCGGCAAATTTGTTCATGG	chr14	64591335	64591357	plus	45	TTS	161	NR_037239	Mir3078	ncRNA	GCGAUCGGCAAAUUUGUUCA	YES	0.2540142	0.131312544	0.926610743	GGCGAUCGGCAAAUUUGUUCAGUUUUAGAGCUAGAAAUAGCAAGUUAAAAUAAGGCUAGUCCGUUAUCAACUUGAAAAAGUGGCACCGAGUCGGUGCUUU	...((((((...(((.(((..((((((.((((....))))...)))))))..))).))))))..))).((((....))))((((((...))))))..	-22.3
50	TOP_17_2_5_1	ATTCCTCGGGAAGGCCCCGCTGG	chr14	63122237	63122259	minus	70	promoter-TSS	214	NM_007798	Ctsb	protein-coding	AUUCCUCGGGAAGGCCCCGC	NO	0.001016575	0.129115108	0.998434908	GAUUCCUCGGGAAGGCCCCGCGUUUUAGAGCUAGAAAUAGCAAGUUAAAAUAAGGCUAGUCCGUUAUCAACUUGAAAAAGUGGCACCGAGUCGGUGCUUU	(((....((((..((((....((((((.((((....))))...)))))))..)))).))))..))).((((....))))((((((...))))))..	-28.2
51	TOP_17_3_1	GAATCTATTACGTACTGCAGAGG	chr8	36147866	36147888	plus	40	TTS	53049	NM_172911	D8Ertd82e	protein-coding	GAAUCUAUUACGUACUGCAG	YES	0.6325076	1.07E-01	0.982043968	GGAAUCUAUUACGUACUGCAGGUUUUAGAGCUAGAAAUAGCAAGUUAAAAUAAGGCUAGUCCGUUAUCAACUUGAAAAAGUGGCACCGAGUCGGUGCUUU	..........(((.(((((..((((((.((((....))))...)))))))...)).))).)))......((((....))))((((((...))))))..	-24.3
52	TOP_17_3_2	CTGCAGAGGAGCGGACGATCTGG	chr8	36147880	36147902	plus	65	TTS	53063	NM_172911	D8Ertd82e	protein-coding	CUGCAGAGGAGCGGACGAUC	NO	0.006047329	1.20E-01	0.99978723	GCUGCAGAGGAGCGGACGAUCGUUUUAGAGCUAGAAAUAGCAAGUUAAAAUAAGGCUAGUCCGUUAUCAACUUGAAAAAGUGGCACCGAGUCGGUGCUUU	......((..((((((..((((((((.((((....))))...)))))))..))...))))))).)).((((....))))((((((...))))))..	-27.9
53	TOP_17_3_3	CCGTATTCAAGTGCAAGTTCTGG	chr8	36147910	36147932	minus	45	TTS	-53093	NM_172911	D8Ertd82e	protein-coding	CCGUAUUCAAGUGCAAGUUC	YES	0.4288153	1.52E-01	0.733429117	GCCGUAUUCAAGUGCAAGUUCGUUUUAGAGCUAGAAAUAGCAAGUUAAAAUAAGGCUAGUCCGUUAUCAACUUGAAAAAGUGGCACCGAGUCGGUGCUUU	......(((((((((.((((.((((((.((((....))))...)))))))..)))).)).........)))))))......((((((...))))))..	-23.3
54	TOP_17_5_1	TCCGTATTTGTAAGCATATCTGG	chr8	34828439	34828461	minus	35	TTS	-20840	NM_176933	Dusp4	protein-coding	UCCGUAUUUGUAAGCAUAUC	NO	0.3622014	1.09E-01	0.676852465	GUCCGUAUUUGUAAGCAUAUCGUUUUAGAGCUAGAAAUAGCAAGUUAAAAUAAGGCUAGUCCGUUAUCAACUUGAAAAAGUGGCACCGAGUCGGUGCUUU	.............(((.....((((((.((((....))))...)))))))...)))............((((....))))((((((...))))))..	-19.8
55	TOP_17_5_2	TAAGCACCCTTCATGCAACTGGG	chr8	34828531	34828553	plus	45	TTS	20932	NM_176933	Dusp4	protein-coding	UAAGCACCCUUCAUGCAACU	YES	0.3997768	1.84E-01	0.985517782	GUAAGCACCCUUCAUGCAACUGUUUUAGAGCUAGAAAUAGCAAGUUAAAAUAAGGCUAGUCCGUUAUCAACUUGAAAAAGUGGCACCGAGUCGGUGCUUU	..((((((((((..(((.(((.(((((..((((....)))).((((...((((((.....)).)))).))))))))))).))).)))..)))..))))))).	-22.1
56	TOP_17_5_3	GCACTAACATCGCCTCCTGAAGG	chr8	34828788	34828810	minus	55	TTS	-21189	NM_176933	Dusp4	protein-coding	GCACUAACAUCGCCUCCUGA	YES	0.3276048	1.19E-01	0.988319902	GGCACUAACAUCGCCUCCUGAGUUUUAGAGCUAGAAAUAGCAAGUUAAAAUAAGGCUAGUCCGUUAUCAACUUGAAAAAGUGGCACCGAGUCGGUGCUUU	((.((((.....((((.....((((((.((((....))))...))))))).))))))))))........((((....))))((((((...))))))..	-24.9
57	TOP_17_5_4	CGAGTCTTATATAGGAATCCAGG	chr8	34829006	34829028	minus	40	TTS	-21407	NM_176933	Dusp4	protein-coding	CGAGUCUUAUAUAGGAAUCC	YES	0.2711322	1.59E-01	0.973659276	GCGAGUCUUAUAUAGGAAUCCGUUUUAGAGCUAGAAAUAGCAAGUUAAAAUAAGGCUAGUCCGUUAUCAACUUGAAAAAGUGGCACCGAGUCGGUGCUUU	.(((((...(((..(((..((((((((.((((....))))...))))))))..))....)))..)))..)))))........((((((...))))))..	-24.8
58	TOP_18_3_1	TCAGGGGTCGGGTTTGCCAAGGG	chr14	54441437	54441459	minus	60	TTS	-9844	NM_008608	Mmp14	protein-coding	UCAGGGGUCGGGUUUGCCAA	YES	0.0477056	0.17160302	0.999976385	GUCAGGGGUCGGGUUUGCCAAGUUUUAGAGCUAGAAAUAGCAAGUUAAAAUAAGGCUAGUCCGUUAUCAACUUGAAAAAGUGGCACCGAGUCGGUGCUUU	.(((((((((((((.(((..((((((.((((....))))...)))))))..))).))))))..)))..)))))......((((((...))))))..	-30.2
59	TOP_18_5_1	ACGACTGATAGGGCCAACAAAGG	chr14	53805842	53805864	minus	50	Intergenic	-139913	NM_001205242	A430107P09Rik	protein-coding	ACGACUGAUAGGGCCAACAA	YES	0.2587349	0.146531408	0.989543293	GACGACUGAUAGGGCCAACAAGUUUUAGAGCUAGAAAUAGCAAGUUAAAAUAAGGCUAGUCCGUUAUCAACUUGAAAAAGUGGCACCGAGUCGGUGCUUU	((.(((.(((..((((.....((((((.((((....))))...)))))))..)))).))).))).)).((((....))))((((((...))))))..	-27.8
60	TOP_19_3_1	AAGCGAGCGGGCGAGTGCCGGGG	chr11	104442330	104442352	plus	75	promoter-TSS	-50	NM_001081045	Kansl1	protein-coding	AAGCGAGCGGGCGAGUGCCG	NO	0.000199648	1.31E-01	0.999843554	GAAGCGAGCGGGCGAGUGCCGGUUUUAGAGCUAGAAAUAGCAAGUUAAAAUAAGGCUAGUCCGUUAUCAACUUGAAAAAGUGGCACCGAGUCGGUGCUUU	......((((((....(((.((((((.((((....))))...)))))))..)))..)))))))....((((....))))((((((...))))))..	-33.8
61	TOP_19_5_1	GACCATGCCAAGTCTTATAAAGG	chr11	103533898	103533920	minus	40	TTS	-80830	NM_001033434	Gm884	protein-coding	GACCAUGCCAAGUCUUAUAA	YES	0.6627541	0.134202997	0.998986297	GGACCAUGCCAAGUCUUAUAAGUUUUAGAGCUAGAAAUAGCAAGUUAAAAUAAGGCUAGUCCGUUAUCAACUUGAAAAAGUGGCACCGAGUCGGUGCUUU	((((.......(((((((....(((((.((((....))))...)))))))))))))).))))........((((....))))((((((...))))))..	-25.4
62	Top_2_5_1	GCTGGATGCGGTGCGTCCGTGGG	chr7	55794007	55794029	minus	70	promoter-TSS	130	NM_146190	Tubgcp5	protein-coding	GCUGGAUGCGGUGCGUCCGU	NO	0.00131022	1.36E-01	0.935651039	GGCUGGAUGCGGUGCGUCCGUGUUUUAGAGCUAGAAAUAGCAAGUUAAAAUAAGGCUAGUCCGUUAUCAACUUGAAAAAGUGGCACCGAGUCGGUGCUUU	.....((((((.((..((.((((((((.((((....))))...)))))))).))...))))).)))).((((....))))((((((...))))))..	-29.3
63	TOP_20_3_1	CCATCAGCCTCGGCGCTCGGGGG	chr11	76763628	76763650	minus	75	promoter-TSS	84	NM_016810	Gosr1	protein-coding	CCAUCAGCCUCGGCGCUCGG	NO	0.001276963	6.08E-02	0.771909219	GCCAUCAGCCUCGGCGCUCGGGUUUUAGAGCUAGAAAUAGCAAGUUAAAAUAAGGCUAGUCCGUUAUCAACUUGAAAAAGUGGCACCGAGUCGGUGCUUU	(((..........)))((((.........))))..........((((((...((((((.....)).)))).))))))........((((((...))))))..	-25.2
64	TOP_20_5_1	GCTTGCGTGGTTAGTAGCGCAGG	chr11	73329642	73329664	plus	60	promoter-TSS	-88	NM_001045531	Spata22	protein-coding	GCUUGCGUGGUUAGUAGCGC	YES	0.1048724	0.138874342	0.865984925	GGCUUGCGUGGUUAGUAGCGCGUUUUAGAGCUAGAAAUAGCAAGUUAAAAUAAGGCUAGUCCGUUAUCAACUUGAAAAAGUGGCACCGAGUCGGUGCUUU	((((((((((.........)))))....)))))).........((((((...((((((.....)).)))).))))))........((((((...))))))..	-25.6
65	TOP_21_3_1	GTCTACTTAGAGCGTAATCATGG	chr8	46847485	46847507	plus	40	TTS	107751	NM_008391	Irf2	protein-coding	GUCUACUUAGAGCGUAAUCA	YES	0.1045511	1.38E-01	0.898082654	GGUCUACUUAGAGCGUAAUCAGUUUUAGAGCUAGAAAUAGCAAGUUAAAAUAAGGCUAGUCCGUUAUCAACUUGAAAAAGUGGCACCGAGUCGGUGCUUU	..((((((((((((........))))).))).))))......((((((...((((((.....)).)))).))))))........((((((...))))))..	-22
66	TOP_21_5_1	GACACACATTCCCGGCGCTGTGG	chr8	41397269	41397291	minus	65	TTS	-19838	NM_013522	Frg1	protein-coding	GACACACAUUCCCGGCGCUG	NO	0.1371866	8.91E-02	0.995022498	GGACACACAUUCCCGGCGCUGGUUUUAGAGCUAGAAAUAGCAAGUUAAAAUAAGGCUAGUCCGUUAUCAACUUGAAAAAGUGGCACCGAGUCGGUGCUUU	(((.......)))(((.((((((((((..((((....)))).........)))))))))))))......((((....))))((((((...))))))..	-27.5
67	TOP_21_5_2	GGTTAACGTTGGCCCTTCACGGG	chr8	41397370	41397392	minus	55	TTS	-19737	NM_013522	Frg1	protein-coding	GGUUAACGUUGGCCCUUCAC	YES	0.1148255	1.69E-01	0.991249966	GGGUUAACGUUGGCCCUUCACGUUUUAGAGCUAGAAAUAGCAAGUUAAAAUAAGGCUAGUCCGUUAUCAACUUGAAAAAGUGGCACCGAGUCGGUGCUUU	.(((.((((((((((.((...((((((.((((....))))...))))))))))))))))..))))))).((((....))))((((((...))))))..	-26.6
68	TOP_21_5_3	TGGCTGGTCATGGTTAACGTTGG	chr8	41397381	41397403	minus	50	TTS	-19726	NM_013522	Frg1	protein-coding	UGGCUGGUCAUGGUUAACGU	YES	0.01609666	1.66E-01	0.991445195	GUGGCUGGUCAUGGUUAACGUGUUUUAGAGCUAGAAAUAGCAAGUUAAAAUAAGGCUAGUCCGUUAUCAACUUGAAAAAGUGGCACCGAGUCGGUGCUUU	..((((((((.((.(((((.((((...............)))).)))))..)).))))))))........((((....))))((((((...))))))..	-26.3
69	TOP_21_5_4	GACCGATACTGCAAGTGACGTGG	chr8	41397603	41397625	minus	55	exon	-19504	NM_013522	Frg1	protein-coding	GACCGAUACUGCAAGUGACG	YES	0.06157276	5.95E-02	0.76202057	GGACCGAUACUGCAAGUGACGGUUUUAGAGCUAGAAAUAGCAAGUUAAAAUAAGGCUAGUCCGUUAUCAACUUGAAAAAGUGGCACCGAGUCGGUGCUUU	.(((((.((((...)))).)))))......((((....))))(((((...((((((.....)).)))).)))))........((((((...))))))..	-23.3
70	TOP_22_3_1	GCTGGGCTAGGTTGTCTACCTGG	chr5	86888874	86888896	plus	60	TTS	18052	NM_153598	Ugt2b34	protein-coding	GCUGGGCUAGGUUGUCUACC	YES	0.01082248	1.39E-01	0.997714257	GGCUGGGCUAGGUUGUCUACCGUUUUAGAGCUAGAAAUAGCAAGUUAAAAUAAGGCUAGUCCGUUAUCAACUUGAAAAAGUGGCACCGAGUCGGUGCUUU	(((.((((((....((((...((((((.((((....))))...))))))).))))))))))))....((((....))))((((((...))))))..	-28
71	TOP_23_3_1	GCATCTGGATTTCACCGAGCTGG	chr5	87492122	87492144	minus	55	Intergenic	1262	NM_053184	Ugt2a1	protein-coding	GCAUCUGGAUUUCACCGAGC	YES	0.2253625	1.01E-01	0.948055668	GGCAUCUGGAUUUCACCGAGCGUUUUAGAGCUAGAAAUAGCAAGUUAAAAUAAGGCUAGUCCGUUAUCAACUUGAAAAAGUGGCACCGAGUCGGUGCUUU	......((((((...((.....((((((.((((....))))...)))))))..))..))))))......((((....))))((((((...))))))..	-24
72	TOP_23_5_1	GCCCTGGCGTTCCCCTGTACTGG	chr1	3014857	3014879	plus	70	Intergenic	656630	NM_001011874	Xkr4	protein-coding	GCCCUGGCGUUCCCCUGUAC	NO	0.1066303	1.08E-01	0.86996374	GGCCCUGGCGUUCCCCUGUACGUUUUAGAGCUAGAAAUAGCAAGUUAAAAUAAGGCUAGUCCGUUAUCAACUUGAAAAAGUGGCACCGAGUCGGUGCUUU	((..(((((.((..........((((((.((((....))))...))))))))).))))).)).......((((....))))((((((...))))))..	-25
73	TOP_23_5_2	AGTCAAGAGCTCCGGGGTACTGG	chr1	3087869	3087891	plus	60	Intergenic	583618	NM_001011874	Xkr4	protein-coding	AGUCAAGAGCUCCGGGGUAC	YES	0.1658467	8.81E-02	0.987306294	GAGUCAAGAGCUCCGGGGUACGUUUUAGAGCUAGAAAUAGCAAGUUAAAAUAAGGCUAGUCCGUUAUCAACUUGAAAAAGUGGCACCGAGUCGGUGCUUU	((((.....))))((((.((.((((((..((((....)))).........))))))).))))......((((....))))((((((...))))))..	-25.5
74	TOP_23_5_3	CCCCAGTACAGGGGAACGCCAGG	chr1	3091547	3091569	plus	70	Intergenic	579940	NM_001011874	Xkr4	protein-coding	CCCCAGUACAGGGGAACGCC	NO	0.01278896	4.13E-02	0.992062616	GCCCCAGUACAGGGGAACGCCGUUUUAGAGCUAGAAAUAGCAAGUUAAAAUAAGGCUAGUCCGUUAUCAACUUGAAAAAGUGGCACCGAGUCGGUGCUUU	.((((......))))...((((((((((.((((....))))...)))))))..)))..............((((....))))((((((...))))))..	-29.3
75	TOP_23_5_4	CTTTGCAGTGATGGCCGACTAGG	chr1	3290904	3290926	plus	55	intron	380583	NM_001011874	Xkr4	protein-coding	CUUUGCAGUGAUGGCCGACU	YES	0.08988987	2.29E-01	0.998557266	GCUUUGCAGUGAUGGCCGACUGUUUUAGAGCUAGAAAUAGCAAGUUAAAAUAAGGCUAGUCCGUUAUCAACUUGAAAAAGUGGCACCGAGUCGGUGCUUU	(((((.((((((((((.((((((((((..((((....)))).........)))))).)))).)))))))..)))..)))))((((((...))))))..	-30.7
76	TOP_24_2_1	GTGTATTAGTCCCTGTAGGCTGG	chr7	102078536	102078558	plus	50	intron	-4569	NM_001109897	Trpc2	protein-coding	GUGUAUUAGUCCCUGUAGGC	YES	0.09415714	0.111949945	0.925489183	GGUGUAUUAGUCCCUGUAGGCGUUUUAGAGCUAGAAAUAGCAAGUUAAAAUAAGGCUAGUCCGUUAUCAACUUGAAAAAGUGGCACCGAGUCGGUGCUUU	((...(((((((((....)).((((((.((((....))))...)))))))..)))))))))).......((((....))))((((((...))))))..	-22
77	TOP_24_2_2	GAGACTTAACCCCTCCGTTCTGG	chr7	102238630	102238652	minus	55	TTS	-11477	NM_019566	Rhog	protein-coding	GAGACUUAACCCCUCCGUUC	YES	0.5130001	4.69E-02	0.887612494	GGAGACUUAACCCCUCCGUUCGUUUUAGAGCUAGAAAUAGCAAGUUAAAAUAAGGCUAGUCCGUUAUCAACUUGAAAAAGUGGCACCGAGUCGGUGCUUU	((((..........))))((((.......))))..........((((((...((((((.....)).)))).))))))........((((((...))))))..	-24
78	TOP_24_3_1	AGAGCCCGCGAGGTCATTGCTGG	chr7	143784519	143784541	minus	65	promoter-TSS	30	NM_175534	Mrgpre	protein-coding	AGAGCCCGCGAGGUCAUUGC	NO	0.03719515	7.70E-02	0.949686525	GAGAGCCCGCGAGGUCAUUGCGUUUUAGAGCUAGAAAUAGCAAGUUAAAAUAAGGCUAGUCCGUUAUCAACUUGAAAAAGUGGCACCGAGUCGGUGCUUU	...((((((((.....)))))((((((.((((....))))...))))))...))))............((((....))))((((((...))))))..	-24.7
79	TOP_24_5_1	GATGGAAACGCGGTGTAATATGG	chr7	143688693	143688715	minus	45	TTS	2832	NM_024290	Tnfrsf23	protein-coding	GAUGGAAACGCGGUGUAAUA	YES	0.4727593	1.17E-01	0.988315484	GGAUGGAAACGCGGUGUAAUAGUUUUAGAGCUAGAAAUAGCAAGUUAAAAUAAGGCUAGUCCGUUAUCAACUUGAAAAAGUGGCACCGAGUCGGUGCUUU	.((((.....((((.....((((((((..((((....)))).........))))))))..)))))))).((((....))))((((((...))))))..	-24.2
80	TOP_25_3_1	CGGTGAAGTGTCTAGGCCGCTGG	chr6	129275472	129275494	minus	65	promoter-TSS	114	NM_001033122	Cd69	protein-coding	CGGUGAAGUGUCUAGGCCGC	NO	0.076075	1.57E-01	0.997925422	GCGGUGAAGUGUCUAGGCCGCGUUUUAGAGCUAGAAAUAGCAAGUUAAAAUAAGGCUAGUCCGUUAUCAACUUGAAAAAGUGGCACCGAGUCGGUGCUUU	..(((((.....((((.((..((((((.((((....))))...)))))))..))))))....))))).((((....))))((((((...))))))..	-29
81	TOP_25_5_1	GCAGTTTGAATGAGAACCGCTGG	chr6	129267300	129267322	plus	50	TTS	8058	NM_001033122	Cd69	protein-coding	GCAGUUUGAAUGAGAACCGC	YES	0.1246711	1.06E-01	0.720224095	GGCAGUUUGAAUGAGAACCGCGUUUUAGAGCUAGAAAUAGCAAGUUAAAAUAAGGCUAGUCCGUUAUCAACUUGAAAAAGUGGCACCGAGUCGGUGCUUU	.((.((((......)))).))((((((.((((....))))...)))))))..................((((....))))((((((...))))))..	-21.9
82	TOP_26_3_1	CCCCATCCACTAGTATAGGTAGG	chr12	113525757	113525779	plus	50	Intergenic	-18140	NM_174885	Adam6a	protein-coding	CCCCAUCCACUAGUAUAGGU	YES	0.05623759	8.98E-02	0.997399357	GCCCCAUCCACUAGUAUAGGUGUUUUAGAGCUAGAAAUAGCAAGUUAAAAUAAGGCUAGUCCGUUAUCAACUUGAAAAAGUGGCACCGAGUCGGUGCUUU	.........((((((.....((((((((.((((....))))...))))))))..))))))..........((((....))))((((((...))))))..	-24.8
83	TOP_26_5_1	AACAGGACTGTTCATCGCCCCGG	chr12	111828241	111828263	minus	55	TTS	-14082	NM_026752	Zfyve21	protein-coding	AACAGGACUGUUCAUCGCCC	YES	0.3718641	0.143320051	0.999609096	GAACAGGACUGUUCAUCGCCCGUUUUAGAGCUAGAAAUAGCAAGUUAAAAUAAGGCUAGUCCGUUAUCAACUUGAAAAAGUGGCACCGAGUCGGUGCUUU	.(((.((((((......(((.((((((.((((....))))...))))))))..)))))))))))....((((....))))((((((...))))))..	-29.3
84	TOP_27_2_1	AGATTGAGACCGCCCGCATCAGG	chr17	23550698	23550720	plus	60	Intergenic	13517	NM_001033496	Zfp213	protein-coding	AGAUUGAGACCGCCCGCAUC	YES	0.3532097	1.31E-01	0.99607766	GAGAUUGAGACCGCCCGCAUCGUUUUAGAGCUAGAAAUAGCAAGUUAAAAUAAGGCUAGUCCGUUAUCAACUUGAAAAAGUGGCACCGAGUCGGUGCUUU	((...((.(((.(((......((((((.((((....))))...)))))))..)))..)))))...)).((((....))))((((((...))))))..	-24
85	TOP_27_2_2	CTGTATGGCCTACAAACCCGAGG	chr17	26285674	26285696	plus	55	TTS	32775	NM_025881	Luc7l	protein-coding	CUGUAUGGCCUACAAACCCG	YES	0.07924306	0.131542763	0.662225986	GCUGUAUGGCCUACAAACCCGGUUUUAGAGCUAGAAAUAGCAAGUUAAAAUAAGGCUAGUCCGUUAUCAACUUGAAAAAGUGGCACCGAGUCGGUGCUUU	(((.((.((((.........)))).)).)))..........((((((...((((((.....)).)))).))))))........((((((...))))))..	-24
86	TOP_27_3_1	GATTTGTACCTCGATGTCATAGG	chr11	32288675	32288697	minus	40	TTS	-1722	NM_001033981	Hbq1b	protein-coding	GAUUUGUACCUCGAUGUCAU	YES	0.5316903	0.109540824	0.974917374	GGAUUUGUACCUCGAUGUCAUGUUUUAGAGCUAGAAAUAGCAAGUUAAAAUAAGGCUAGUCCGUUAUCAACUUGAAAAAGUGGCACCGAGUCGGUGCUUU	(((((....(((.........((((((.((((....))))...)))))))))))..)))))........((((....))))((((((...))))))..	-24
87	TOP_27_5_1	GCTCAGGGGTCTCCGCTGTTGGG	chr11	32288651	32288673	plus	65	TTS	1698	NM_001033981	Hbq1b	protein-coding	GCUCAGGGGUCUCCGCUGUU	NO	0.05185343	1.23E-01	0.957397412	GGCUCAGGGGUCUCCGCUGUUGUUUUAGAGCUAGAAAUAGCAAGUUAAAAUAAGGCUAGUCCGUUAUCAACUUGAAAAAGUGGCACCGAGUCGGUGCUUU	.....((.((.((..(((.((((((((.((((....))))...))))))))))))).)).)).))....((((....))))((((((...))))))..	-26.7
88	TOP_28_3_1	ACAGGCTTATTGCATCGTATAGG	chr5	132802000	132802022	plus	40	Intergenic	-259668	NM_177047	Auts2	protein-coding	ACAGGCUUAUUGCAUCGUAU	YES	0.1612472	1.47E-01	0.971084745	GACAGGCUUAUUGCAUCGUAUGUUUUAGAGCUAGAAAUAGCAAGUUAAAAUAAGGCUAGUCCGUUAUCAACUUGAAAAAGUGGCACCGAGUCGGUGCUUU	(((((.((((((((((...))))......((((....)))).......)))))).)).)))........((((....))))((((((...))))))..	-23.9
89	TOP_28_5_1	AAGAAGCACACGGAGGTCGCAGG	chr5	130378423	130378445	minus	60	TTS	-6197	NM_198665	A330070K13Rik	protein-coding	AAGAAGCACACGGAGGUCGC	YES	0.04455295	1.25E-01	0.999233144	GAAGAAGCACACGGAGGUCGCGUUUUAGAGCUAGAAAUAGCAAGUUAAAAUAAGGCUAGUCCGUUAUCAACUUGAAAAAGUGGCACCGAGUCGGUGCUUU	..........((((((((..((((((.((((....))))...)))))))..))))..))))).....((((....))))((((((...))))))..	-28.9
90	TOP_28_5_2	TTAGCTAGGCTAGGCGTTTGTGG	chr5	130378629	130378651	minus	50	TTS	-5991	NM_198665	A330070K13Rik	protein-coding	UUAGCUAGGCUAGGCGUUUG	YES	0.005588457	1.54E-01	0.997663661	GUUAGCUAGGCUAGGCGUUUGGUUUUAGAGCUAGAAAUAGCAAGUUAAAAUAAGGCUAGUCCGUUAUCAACUUGAAAAAGUGGCACCGAGUCGGUGCUUU	..((((..(((((.((.((..((((((.((((....))))...))))))))))).))))))).))))...((((....))))((((((...))))))..	-27.2
91	TOP_28_5_3	TTCTAGAGACCCCCCAATACAGG	chr5	130378801	130378823	minus	50	TTS	-5819	NM_198665	A330070K13Rik	protein-coding	UUCUAGAGACCCCCCAAUAC	YES	0.6908868	1.20E-01	0.984709169	GUUCUAGAGACCCCCCAAUACGUUUUAGAGCUAGAAAUAGCAAGUUAAAAUAAGGCUAGUCCGUUAUCAACUUGAAAAAGUGGCACCGAGUCGGUGCUUU	(((((((((.............)))))))))..........((((((...((((((.....)).)))).))))))........((((((...))))))..	-21.7
92	TOP_29_3_1	ACCTAGTAGATTCCGGACAGAGG	chr8	38661637	38661659	minus	50	promoter-TSS	140	NM_145841	Sgcz	protein-coding	ACCUAGUAGAUUCCGGACAG	YES	0.116917	1.46E-01	0.999958903	GACCUAGUAGAUUCCGGACAGGUUUUAGAGCUAGAAAUAGCAAGUUAAAAUAAGGCUAGUCCGUUAUCAACUUGAAAAAGUGGCACCGAGUCGGUGCUUU	.........(((..(((((.((((((((..((((....)))).........)))))))).)))))..))).((((....))))((((((...))))))..	-28.9
93	TOP_29_5_1	GAATCTTCCTATATTAGCCGAGG	chr8	36567585	36567607	minus	40	TTS	-46347	NM_015802	Dlc1	protein-coding	GAAUCUUCCUAUAUUAGCCG	YES	0.5806252	8.77E-02	0.999970974	GGAAUCUUCCUAUAUUAGCCGGUUUUAGAGCUAGAAAUAGCAAGUUAAAAUAAGGCUAGUCCGUUAUCAACUUGAAAAAGUGGCACCGAGUCGGUGCUUU	(((....)))...(((((((.((((((.((((....))))...)))))))..)))))))..........((((....))))((((((...))))))..	-29.2
94	TOP_30_3_1	ATGCCCCCCATGGCCCCATAAGG	chr16	8922726	8922748	minus	65	Intergenic	-92637	NM_001081400	1810013L24Rik	protein-coding	AUGCCCCCCAUGGCCCCAUA	NO	0.2658758	1.25E-01	0.991571034	GAUGCCCCCCAUGGCCCCAUAGUUUUAGAGCUAGAAAUAGCAAGUUAAAAUAAGGCUAGUCCGUUAUCAACUUGAAAAAGUGGCACCGAGUCGGUGCUUU	((((.......(((((.....((((((.((((....))))...)))))))..)))))......)))).((((....))))((((((...))))))..	-24.8
95	TOP_30_3_2	TAGCACACAGGGCTTAGCGCTGG	chr16	8922831	8922853	plus	60	Intergenic	92742	NM_001081400	1810013L24Rik	protein-coding	UAGCACACAGGGCUUAGCGC	YES	0.0261944	0.134053225	0.999681634	GUAGCACACAGGGCUUAGCGCGUUUUAGAGCUAGAAAUAGCAAGUUAAAAUAAGGCUAGUCCGUUAUCAACUUGAAAAAGUGGCACCGAGUCGGUGCUUU	..........(((((.(((..((((((.((((....))))...)))))))...))))))))).......((((....))))((((((...))))))..	-28.4
96	TOP_30_5_1	GTCGGTCGGAGCTACCAAAATGG	chr16	7069736	7069758	minus	55	promoter-TSS	88	NM_021477	Rbfox1	protein-coding	GUCGGUCGGAGCUACCAAAA	YES	0.03465228	1.51E-01	0.998913412	GGUCGGUCGGAGCUACCAAAAGUUUUAGAGCUAGAAAUAGCAAGUUAAAAUAAGGCUAGUCCGUUAUCAACUUGAAAAAGUGGCACCGAGUCGGUGCUUU	(((....((((.(((((....((((((.((((....))))...)))))))..)).)))))))..))).((((....))))((((((...))))))..	-26.2
97	TOP_31_3_1	AATCTGTGATGTCCGATTCTAGG	chr17	76284659	76284681	plus	40	Intergenic	423647	NR_033456	Gm4710	ncRNA	AAUCUGUGAUGUCCGAUUCU	YES	0.409568	1.17E-01	0.821055126	GAAUCUGUGAUGUCCGAUUCUGUUUUAGAGCUAGAAAUAGCAAGUUAAAAUAAGGCUAGUCCGUUAUCAACUUGAAAAAGUGGCACCGAGUCGGUGCUUU	.......(((((...((((((((((((.((((....))))...))))))).)))...)))...)))))((((....))))((((((...))))))..	-22.4
98	TOP_31_3_2	TAAAGGTACCGTGCGTTTGCTGG	chr17	76284691	76284713	plus	50	Intergenic	423679	NR_033456	Gm4710	ncRNA	UAAAGGUACCGUGCGUUUGC	YES	0.04146572	6.49E-02	0.415118761	GUAAAGGUACCGUGCGUUUGCGUUUUAGAGCUAGAAAUAGCAAGUUAAAAUAAGGCUAGUCCGUUAUCAACUUGAAAAAGUGGCACCGAGUCGGUGCUUU	.....((((.((.((((((..((((((.((((....))))...)))))))).))))..)).)).)))).((((....))))((((((...))))))..	-23.6
99	TOP_31_3_3	ATTAAGTAAGTAGCAACGGCAGG	chr17	76285203	76285225	plus	40	Intergenic	424191	NR_033456	Gm4710	ncRNA	AUUAAGUAAGUAGCAACGGC	YES	0.3860479	1.87E-01	0.981810291	GAUUAAGUAAGUAGCAACGGCGUUUUAGAGCUAGAAAUAGCAAGUUAAAAUAAGGCUAGUCCGUUAUCAACUUGAAAAAGUGGCACCGAGUCGGUGCUUU	..((((((..(((((.((((((((((((.((((....))))...)))))))...))).))..)))))..))))))......((((((...))))))..	-24.9
100	TOP_31_5_1	CAGATGAACAGGGTATCGCAAGG	chr17	74426404	74426426	plus	50	3' UTR	30807	NM_144798	Slc30a6	protein-coding	CAGAUGAACAGGGUAUCGCA	YES	0.3004782	1.35E-01	0.988929637	GCAGAUGAACAGGGUAUCGCAGUUUUAGAGCUAGAAAUAGCAAGUUAAAAUAAGGCUAGUCCGUUAUCAACUUGAAAAAGUGGCACCGAGUCGGUGCUUU	...(((((...(((....((.((((((.((((....))))...)))))))...))...))).))))).((((....))))((((((...))))))..	-23.3
101	TOP_31_5_2	ATTGGCTTGAGAAACGAGTTTGG	chr17	74426509	74426531	minus	40	3' UTR	-30912	NM_144798	Slc30a6	protein-coding	AUUGGCUUGAGAAACGAGUU	YES	0.01551425	0.178451626	0.982228533	GAUUGGCUUGAGAAACGAGUUGUUUUAGAGCUAGAAAUAGCAAGUUAAAAUAAGGCUAGUCCGUUAUCAACUUGAAAAAGUGGCACCGAGUCGGUGCUUU	((((((((....(((..((((((((.......))))))))..)))......))))))))........((((....))))((((((...))))))..	-28.1
102	TOP_32_3_1	AGACCGACTCAAATTTTGATTGG	chr12	30959909	30959931	plus	35	exon	48251	NM_013709	Sh3yl1	protein-coding	AGACCGACUCAAAUUUUGAU	NO	0.1167985	1.60E-01	0.953460699	GAGACCGACUCAAAUUUUGAUGUUUUAGAGCUAGAAAUAGCAAGUUAAAAUAAGGCUAGUCCGUUAUCAACUUGAAAAAGUGGCACCGAGUCGGUGCUUU	((((((((((.........((((((((.((((....))))...))))))))..((((.....(((.......))).....))))...)))))))).))).	-24.1
103	TOP_32_3_2	GGAAAACTTCGAGGTCAAACTGG	chr12	30959938	30959960	plus	45	exon	48280	NM_013709	Sh3yl1	protein-coding	GGAAAACUUCGAGGUCAAAC	YES	0.4277328	1.04E-01	0.671269174	GGGAAAACUUCGAGGUCAAACGUUUUAGAGCUAGAAAUAGCAAGUUAAAAUAAGGCUAGUCCGUUAUCAACUUGAAAAAGUGGCACCGAGUCGGUGCUUU	..((.(((.....((((....((((((.((((....))))...)))))))..))))......))).)).((((....))))((((((...))))))..	-21.2

104	TOP_32_3_3	TTTCTATAGTTGCCATCGACAGG	chr12	30960192	30960214	plus	40	TTS	48534	NM_013709	Sh3yl1	protein-coding	UUUCUAUAGUUGCCAUCGAC	YES	0.1053447	1.16E-01	0.5430468	GUUUCUAUAGUUGCCAUCGACGUUUUAGAGCUAGAAAUAGCAAGUUAAAAUAAGGCUAGUCCGUUAUCAACUUGAAAAAGUGGCACCGAGUCGGUGCUUU	[illegible]	-20.7
105	TOP_32_5_1	TTGTGGGGTGATCGTTTTATTGG	chr12	28553756	28553778	plus	40	TTS	-5430	NM_001177964	Dcdc2c	protein-coding	UUGUGGGGUGAUCGUUUUAU	YES	0.3480382	1.99E-01	0.980355776	GUUGUGGGGUGAUCGUUUUAUGUUUUAGAGCUAGAAAUAGCAAGUUAAAAUAAGGCUAGUCCGUUAUCAACUUGAAAAAGUGGCACCGAGUCGGUGCUUU	[illegible]	-23.2
106	TOP_32_5_2	TCTCTCTTAAAACGGGATTCAGG	chr12	28553850	28553872	minus	40	exon	5524	NM_001177964	Dcdc2c	protein-coding	UCUCUCUUAAAACGGGAUUC	YES	0.01125273	1.33E-01	0.930877535	GUCUCUCUUAAAACGGGAUUCGUUUUAGAGCUAGAAAUAGCAAGUUAAAAUAAGGCUAGUCCGUUAUCAACUUGAAAAAGUGGCACCGAGUCGGUGCUUU	[illegible]	-24.3
107	TOP_32_5_3	GTCAGCCGCCTTCGGCTCAAGGG	chr12	28553929	28553951	minus	65	exon	5603	NM_001177964	Dcdc2c	protein-coding	GUCAGCCGCCUUCGGCUCAA	NO	0.007954613	1.04E-01	0.924037821	GGUCAGCCGCCUUCGGCUCAAGUUUUAGAGCUAGAAAUAGCAAGUUAAAAUAAGGCUAGUCCGUUAUCAACUUGAAAAAGUGGCACCGAGUCGGUGCUUU	[illegible]	-26
108	TOP_33_2_1	ACTGTTCTCCCGACCTTACCAGG	chr2	127838605	127838627	minus	55	Intergenic	6757	NM_009772	Bub1	protein-coding	ACUGUUCUCCCGACCUUACC	YES	0.5699886	1.17E-01	0.956678264	GACUGUUCUCCCGACCUUACCGUUUUAGAGCUAGAAAUAGCAAGUUAAAAUAAGGCUAGUCCGUUAUCAACUUGAAAAAGUGGCACCGAGUCGGUGCUUU	[illegible]	-21.6
109	TOP_33_2_2	CTGTTCTCCCGACCTTACCAGG	chr2	127838605	127838626	minus	55	Intergenic	6756	NM_009772	Bub1	protein-coding	CUGUUCUCCCGACCUUACCA	YES	0.7257539	8.48E-02	0.834040582	GCUGUUCUCCCGACCUUACCAGUUUUAGAGCUAGAAAUAGCAAGUUAAAAUAAGGCUAGUCCGUUAUCAACUUGAAAAAGUGGCACCGAGUCGGUGCUUU	[illegible]	-21.1
110	TOP_33_2_3	ATTGGCTAACCTTTGGCGAAAGG	chr2	129235949	129235971	plus	45	TTS	16991	NM_177847	A730036I17Rik	ncRNA	AUUGGCUAACCUUUGGCGAA	YES	0.129141	1.48E-01	0.993072289	GAUUGGCUAACCUUUGGCGAAGUUUUAGAGCUAGAAAUAGCAAGUUAAAAUAAGGCUAGUCCGUUAUCAACUUGAAAAAGUGGCACCGAGUCGGUGCUUU	[illegible]	-25.8
111	TOP_33_2_4	GCTGCATGCGTAAATTTACGGGG	chr2	129236250	129236272	minus	45	TTS	-17292	NM_177847	A730036I17Rik	ncRNA	GCUGCAUGCGUAAAUUUACG	YES	0.02052677	7.14E-02	0.841007491	GGCUGCAUGCGUAAAUUUACGGUUUUAGAGCUAGAAAUAGCAAGUUAAAAUAAGGCUAGUCCGUUAUCAACUUGAAAAAGUGGCACCGAGUCGGUGCUUU	[illegible]	-23.4
112	TOP_33_3_1	TTCATAGTTTCAAGCGTACCTGG	chr10	59226227	59226249	minus	40	3' UTR	-4316	NM_172939	Sowahc	protein-coding	UUCAUAGUUUCAAGCGUACC	YES	0.2594746	0.15315725	0.765943518	GUUCAUAGUUUCAAGCGUACCGUUUUAGAGCUAGAAAUAGCAAGUUAAAAUAAGGCUAGUCCGUUAUCAACUUGAAAAAGUGGCACCGAGUCGGUGCUUU	[illegible]	-22.6
113	TOP_33_5_1	TGGCCCGCTGTGCCGCTCGAGGG	chr10	58255599	58255621	plus	75	promoter-TSS	84	NM_027375	Gcc2	protein-coding	UGGCCCGCUGUGCCGCUCGA	NO	0.0972956	2.31E-01	0.976382001	GUGGCCCGCUGUGCCGCUCGAGUUUUAGAGCUAGAAAUAGCAAGUUAAAAUAAGGCUAGUCCGUUAUCAACUUGAAAAAGUGGCACCGAGUCGGUGCUUU	[illegible]	-28.9
114	TOP_34_3_1	CACTCTGTCTCCGGCAATCGAGG	chr17	12219548	12219570	minus	60	TTS	-99101	NM_011948	Map3k4	protein-coding	CACUCUGUCUCCGGCAAUCG	YES	0.01835934	7.82E-02	0.983688885	GCACUCUGUCUCCGGCAAUCGGUUUUAGAGCUAGAAAUAGCAAGUUAAAAUAAGGCUAGUCCGUUAUCAACUUGAAAAAGUGGCACCGAGUCGGUGCUUU	[illegible]	-24.4
115	TOP_34_5_1	CCACTGTGGGGCAGCGCCTATGG	chr17	10454763	10454785	plus	70	intron	-1442	NR_077237	A230009B12Rik	ncRNA	CCACUGUGGGGCAGCGCCUA	NO	0.000757371	1.25E-01	0.992373443	GCCACUGUGGGGCAGCGCCUAGUUUUAGAGCUAGAAAUAGCAAGUUAAAAUAAGGCUAGUCCGUUAUCAACUUGAAAAAGUGGCACCGAGUCGGUGCUUU	[illegible]	-30.4
116	TOP_35_3_1	CGGTCACGTGTTAGGCCGGAAGG	chr17	15527311	15527333	plus	65	promoter-TSS	-21	NM_008799	Pdcd2	protein-coding	CGGUCACGUGUUAGGCCGGA	NO	0.1968378	1.41E-01	0.992946792	GCGGUCACGUGUUAGGCCGGAGUUUUAGAGCUAGAAAUAGCAAGUUAAAAUAAGGCUAGUCCGUUAUCAACUUGAAAAAGUGGCACCGAGUCGGUGCUUU	[illegible]	-27.2
117	TOP_35_3_2	ATCTCGAGCTGGGACCGGACTGG	chr17	15527350	15527372	plus	65	promoter-TSS	-60	NM_008799	Pdcd2	protein-coding	AUCUCGAGCUGGGACCGGAC	NO	0.02804937	1.64E-01	0.999099401	GAUCUCGAGCUGGGACCGGACGUUUUAGAGCUAGAAAUAGCAAGUUAAAAUAAGGCUAGUCCGUUAUCAACUUGAAAAAGUGGCACCGAGUCGGUGCUUU	[illegible]	-29.1
118	TOP_35_5_1	TAGTGACATTAGCCGCCTGCAGG	chr17	13716547	13716569	plus	55	3' UTR	-43983	NM_010806	Mllt4	protein-coding	UAGUGACAUUAGCCGCCUGC	YES	0.2401871	0.148874799	0.999791447	GUAGUGACAUUAGCCGCCUGCGUUUUAGAGCUAGAAAUAGCAAGUUAAAAUAAGGCUAGUCCGUUAUCAACUUGAAAAAGUGGCACCGAGUCGGUGCUUU	[illegible]	-27.7
119	TOP_35_5_2	TGACATCACATATCGCTGACAGG	chr17	13716641	13716663	minus	45	3' UTR	43889	NM_010806	Mllt4	protein-coding	UGACAUCACAUAUCGCUGAC	YES	0.3066107	4.80E-02	0.858731513	GUGACAUCACAUAUCGCUGACGUUUUAGAGCUAGAAAUAGCAAGUUAAAAUAAGGCUAGUCCGUUAUCAACUUGAAAAAGUGGCACCGAGUCGGUGCUUU	[illegible]	-23.1
120	TOP_36_3_1	TGAGGCTAGCTCGGTATACCTGG	chr6	43666679	43666701	minus	55	promoter-TSS	515	NM_013861	Tpk1	protein-coding	UGAGGCUAGCUCGGUAUACC	YES	0.01909745	0.128612088	0.979941248	GUGAGGCUAGCUCGGUAUACCGUUUUAGAGCUAGAAAUAGCAAGUUAAAAUAAGGCUAGUCCGUUAUCAACUUGAAAAAGUGGCACCGAGUCGGUGCUUU	[illegible]	-27.4
121	TOP_36_5_1	AATGAAGAACTATCTTCGATTGG	chr6	42587171	42587193	minus	30	TTS	-10190	NM_203396	Fam115e	protein-coding	AAUGAAGAACUAUCUUCGAU	NO	0.07185772	1.03E-01	0.843610301	GAAUGAAGAACUAUCUUCGAUGUUUUAGAGCUAGAAAUAGCAAGUUAAAAUAAGGCUAGUCCGUUAUCAACUUGAAAAAGUGGCACCGAGUCGGUGCUUU	[illegible]	-22.5
122	TOP_37_3_1	GGGGAGCGAGCGCCGAAGACAGG	chr8	17535437	17535459	plus	75	promoter-TSS	-63	NM_053171	Csmd1	protein-coding	GGGGAGCGAGCGCCGAAGAC	NO	0.02512754	1.22E-01	0.901892101	GGGGGAGCGAGCGCCGAAGACGUUUUAGAGCUAGAAAUAGCAAGUUAAAAUAAGGCUAGUCCGUUAUCAACUUGAAAAAGUGGCACCGAGUCGGUGCUUU	[illegible]	-24.5
123	TOP_37_5_1	CATTCGTTATGGTGCTAAGCAGG	chr8	15227160	15227182	plus	45	Intergenic	169518	NM_008664	Myom2	protein-coding	CAUUCGUUAUGGUGCUAAGC	YES	0.1082587	1.53E-01	0.790335529	GCAUUCGUUAUGGUGCUAAGCGUUUUAGAGCUAGAAAUAGCAAGUUAAAAUAAGGCUAGUCCGUUAUCAACUUGAAAAAGUGGCACCGAGUCGGUGCUUU	[illegible]	-24.9
124	TOP_38_3_1	CCTCTCGTGAACTGCCCGGTTGG	chr2	28552837	28552859	plus	65	3' UTR	10514	NM_009885	Cel	protein-coding	CCUCUCGUGAACUGCCCGGU	NO	0.1224258	1.16E-01	0.998354456	GCCUCUCGUGAACUGCCCGGUGUUUUAGAGCUAGAAAUAGCAAGUUAAAAUAAGGCUAGUCCGUUAUCAACUUGAAAAAGUGGCACCGAGUCGGUGCUUU	[illegible]	-25.7
125	TOP_38_5_1	AGTGTGTCTCAGTGCGTCGGTGG	chr2	24603833	24603855	minus	60	TTS	128245	NM_011040	Pax8	protein-coding	AGUGUGUCUCAGUGCGUCGG	YES	0.2015348	8.92E-02	0.867354116	GAGUGUGUCUCAGUGCGUCGGGUUUUAGAGCUAGAAAUAGCAAGUUAAAAUAAGGCUAGUCCGUUAUCAACUUGAAAAAGUGGCACCGAGUCGGUGCUUU	[illegible]	-23.4
126	TOP_39_3_1	AAGGTGCACTTCGTGTACTTGGG	chr7	140297793	140297815	plus	45	TTS	-24897	NM_146956	Olfr525	protein-coding	AAGGUGCACUUCGUGUACUU	YES	0.1454874	4.29E-02	0.833361147	GAAGGUGCACUUCGUGUACUUGUUUUAGAGCUAGAAAUAGCAAGUUAAAAUAAGGCUAGUCCGUUAUCAACUUGAAAAAGUGGCACCGAGUCGGUGCUUU	[illegible]	-26.7
127	TOP_39_5_1	CTATGTCTCAGCGGGTAAACTGG	chr7	136894221	136894243	plus	50	promoter-TSS	-379	NM_008598	Mgmt	protein-coding	CUAUGUCUCAGCGGGUAAAC	YES	0.030399	1.53E-01	0.829724392	GCUAUGUCUCAGCGGGUAAACGUUUUAGAGCUAGAAAUAGCAAGUUAAAAUAAGGCUAGUCCGUUAUCAACUUGAAAAAGUGGCACCGAGUCGGUGCUUU	[illegible]	-23.4
128	TOP_40_3_1	TGCATATTAGCTGCCCCAATGGG	chr14	50321017	50321039	minus	45	promoter-TSS	195	NM_146664	Olfr734	protein-coding	UGCAUAUUAGCUGCCCCAAU	YES	0.5057438	0.114063359	0.993266804	GUGCAUAUUAGCUGCCCCAAUGUUUUAGAGCUAGAAAUAGCAAGUUAAAAUAAGGCUAGUCCGUUAUCAACUUGAAAAAGUGGCACCGAGUCGGUGCUUU	[illegible]	-23.5
129	TOP_40_3_2	TCATGCAGGATTCGGTTTCCTGG	chr7	65636380	65636402	minus	50	Intergenic	8507	NM_172310	Tarsl2	protein-coding	UCAUGCAGGAUUCGGUUUCC	YES	0.01374749	0.13800306	0.964793493	GUCAUGCAGGAUUCGGUUUCCGUUUUAGAGCUAGAAAUAGCAAGUUAAAAUAAGGCUAGUCCGUUAUCAACUUGAAAAAGUGGCACCGAGUCGGUGCUUU	[illegible]	-25.5
130	TOP_40_5_1	GAGGAATGCTCCACGAGGTCAGG	chr14	50280979	50281001	minus	60	TTS	-1281	NM_146665	Olfr732	protein-coding	GAGGAAUGCUCCACGAGGUC	YES	0.03201801	6.92E-02	0.89769947	GGAGGAAUGCUCCACGAGGUCGUUUUAGAGCUAGAAAUAGCAAGUUAAAAUAAGGCUAGUCCGUUAUCAACUUGAAAAAGUGGCACCGAGUCGGUGCUUU	[illegible]	-26.5
131	TOP_40_5_2	CATCTTAGGCGCTGTGATAGTGG	chr14	50320591	50320613	plus	50	exon	231	NM_146664	Olfr734	protein-coding	CAUCUUAGGCGCUGUGAUAG	YES	0.1076794	9.52E-02	0.565951357	GCAUCUUAGGCGCUGUGAUAGGUUUUAGAGCUAGAAAUAGCAAGUUAAAAUAAGGCUAGUCCGUUAUCAACUUGAAAAAGUGGCACCGAGUCGGUGCUUU	[illegible]	-21.9
132	TOP_40_5_3	GATTTATACCCTTCGGAATCAGG	chr14	50281375	50281397	minus	40	exon	-885	NM_146665	Olfr732	protein-coding	GAUUUAUACCCUUCGGAAUC	YES	0.253691	1.06E-01	0.993714785	GGAUUUAUACCCUUCGGAAUCGUUUUAGAGCUAGAAAUAGCAAGUUAAAAUAAGGCUAGUCCGUUAUCAACUUGAAAAAGUGGCACCGAGUCGGUGCUUU	[illegible]	-23.3
133	TOP_41_2_3_1	AGGCTTGGCATCCGTAGACGTGG	chr16	14473912	14473934	minus	60	3' UTR	88394	NR_033556	A630010A05Rik	ncRNA	AGGCUUGGCAUCCGUAGACG	YES	0.0348639	8.32E-02	0.909197876	GAGGCUUGGCAUCCGUAGACGGUUUUAGAGCUAGAAAUAGCAAGUUAAAAUAAGGCUAGUCCGUUAUCAACUUGAAAAAGUGGCACCGAGUCGGUGCUUU	[illegible]	-24.6
134	TOP_41_2_5_1	GCTCCAAAGTGTCAATGTACTGG	chr16	13940424	13940446	minus	45	promoter-TSS	194	NM_033564	Mpv17l	protein-coding	GCUCCAAAGUGUCAAUGUAC	YES	0.4201426	1.19E-01	0.997253897	GGCUCCAAAGUGUCAAUGUACGUUUUAGAGCUAGAAAUAGCAAGUUAAAAUAAGGCUAGUCCGUUAUCAACUUGAAAAAGUGGCACCGAGUCGGUGCUUU	[illegible]	-23.9
135	TOP_41_2_5_2	CTTACTTTTTATTGCGGTAGTGG	chr16	13940458	13940480	plus	35	promoter-TSS	-160	NM_033564	Mpv17l	protein-coding	CUUACUUUUUAUUGCGGUAG	NO	0.2751679	1.52E-01	0.92383464	GCUUACUUUUUAUUGCGGUAGGUUUUAGAGCUAGAAAUAGCAAGUUAAAAUAAGGCUAGUCCGUUAUCAACUUGAAAAAGUGGCACCGAGUCGGUGCUUU	[illegible]	-22.8
136	TOP_41_2_5_3	TTACTTTTTATTGCGGTAGTGGG	chr16	13940459	13940481	plus	30	promoter-TSS	-159	NM_033564	Mpv17l	protein-coding	UUACUUUUUAUUGCGGUAGU	NO	0.2697867	1.85E-01	0.990399083	GUUACUUUUUAUUGCGGUAGUGUUUUAGAGCUAGAAAUAGCAAGUUAAAAUAAGGCUAGUCCGUUAUCAACUUGAAAAAGUGGCACCGAGUCGGUGCUUU	[illegible]	-24.6
137	TOP_41_2_5_4	TACTTTTTATTGCGGTAGTGGGG	chr16	13940460	13940482	plus	35	promoter-TSS	-158	NM_033564	Mpv17l	protein-coding	UACUUUUUAUUGCGGUAGUG	NO	0.1445722	1.75E-01	0.998602054	GUACUUUUUAUUGCGGUAGUGGUUUUAGAGCUAGAAAUAGCAAGUUAAAAUAAGGCUAGUCCGUUAUCAACUUGAAAAAGUGGCACCGAGUCGGUGCUUU	[illegible]	-25
138	TOP_41_2_5_5	GGTGATGTACGGCGTCCACCGGG	chr16	13940552	13940574	minus	65	promoter-TSS	66	NM_033564	Mpv17l	protein-coding	GGUGAUGUACGGCGUCCACC	NO	0.001601614	1.55E-01	0.977583409	GGGUGAUGUACGGCGUCCACCGUUUUAGAGCUAGAAAUAGCAAGUUAAAAUAAGGCUAGUCCGUUAUCAACUUGAAAAAGUGGCACCGAGUCGGUGCUUU	[illegible]	-26.3
139	TOP_41_2_5_6	TGGTGATGTACGGCGTCCACCGG	chr16	13940553	13940575	minus	60	promoter-TSS	65	NM_033564	Mpv17l	protein-coding	UGGUGAUGUACGGCGUCCAC	YES	0.01974254	1.57E-01	0.997316358	GUGGUGAUGUACGGCGUCCACGUUUUAGAGCUAGAAAUAGCAAGUUAAAAUAAGGCUAGUCCGUUAUCAACUUGAAAAAGUGGCACCGAGUCGGUGCUUU	[illegible]	-28.2
140	TOP_41_3_1	GTAATGATGGAGGAACTCGCAGG	chr16	13949825	13949847	plus	50	TTS	9171	NM_033564	Mpv17l	protein-coding	GUAAUGAUGGAGGAACUCGC	YES	0.04329323	1.62E-01	0.999677496	GGUAAUGAUGGAGGAACUCGCGUUUUAGAGCUAGAAAUAGCAAGUUAAAAUAAGGCUAGUCCGUUAUCAACUUGAAAAAGUGGCACCGAGUCGGUGCUUU	[illegible]	-29.5
141	TOP_41_3_3_1	AAGATACAGGACGTACATCCAGG	chr7	46084327	46084349	minus	45	TTS	-15899	NM_010602	Kcnj11	protein-coding	AAGAUACAGGACGUACAUCC	YES	0.01682248	1.24E-01	0.999552127	GAAGAUACAGGACGUACAUCCGUUUUAGAGCUAGAAAUAGCAAGUUAAAAUAAGGCUAGUCCGUUAUCAACUUGAAAAAGUGGCACCGAGUCGGUGCUUU	[illegible]	-26.8
142	TOP_41_3_5_1	AACGCAGTTGTTAAGAGCGATGG	chr7	45976194	45976216	plus	45	TTS	47807	NM_001033243	Ccdc114	protein-coding	AACGCAGUUGUUAAGAGCGA	YES	0.06793482	1.43E-01	0.929039842	GAACGCAGUUGUUAAGAGCGAGUUUUAGAGCUAGAAAUAGCAAGUUAAAAUAAGGCUAGUCCGUUAUCAACUUGAAAAAGUGGCACCGAGUCGGUGCUUU	[illegible]	-21.9
143	TOP_41_5_1	AGTTGAGCCGAACAATATGCAGG	chr16	13714662	13714684	plus	45	TTS	-15163	NR_040574	Pla2g10os	ncRNA	AGUUGAGCCGAACAAUAUGC	YES	0.3153088	0.153298418	0.994540801	GAGUUGAGCCGAACAAUAUGCGUUUUAGAGCUAGAAAUAGCAAGUUAAAAUAAGGCUAGUCCGUUAUCAACUUGAAAAAGUGGCACCGAGUCGGUGCUUU	[illegible]	-24.3
144	TOP_42_3_1	GCCCCGACCTTGCCATTAACTGG	chr10	75586410	75586432	plus	60	TTS	-2960	NM_011820	Ggt5	protein-coding	GCCCCGACCUUGCCAUUAAC	YES	0.6905254	0.191037734	0.965056195	GGCCCCGACCUUGCCAUUAACGUUUUAGAGCUAGAAAUAGCAAGUUAAAAUAAGGCUAGUCCGUUAUCAACUUGAAAAAGUGGCACCGAGUCGGUGCUUU	[illegible]	-24
145	TOP_42_3_2	AAGTAATCGGCATCATCCTCCGG	chr10	75949409	75949431	minus	45	exon	-1108	NM_009514	Vpreb3	protein-coding	AAGUAAUCGGCAUCAUCCUC	YES	0.332749	0.142536124	0.999570999	GAAGUAAUCGGCAUCAUCCUCGUUUUAGAGCUAGAAAUAGCAAGUUAAAAUAAGGCUAGUCCGUUAUCAACUUGAAAAAGUGGCACCGAGUCGGUGCUUU	[illegible]	-25.9
146	TOP_42_5_1	CCAGGAACATGAGGCGGCACGGG	chr10	74957445	74957467	plus	65	TTS	-9775	NM_010311	Gnaz	protein-coding	CCAGGAACAUGAGGCGGCAC	NO	0.09301604	0.132291707	0.928102896	GCCAGGAACAUGAGGCGGCACGUUUUAGAGCUAGAAAUAGCAAGUUAAAAUAAGGCUAGUCCGUUAUCAACUUGAAAAAGUGGCACCGAGUCGGUGCUUU	[illegible]	-24.8
147	TOP_42_5_2	CCAACGCTCTTCCCGCGGCGCGG	chr10	75060485	75060507	plus	75	promoter-TSS	-400	NM_001081412	Bcr	protein-coding	CCAACGCUCUUCCCGCGGCG	NO	0.01582595	5.56E-02	0.483016171	GCCAACGCUCUUCCCGCGGCGGUUUUAGAGCUAGAAAUAGCAAGUUAAAAUAAGGCUAGUCCGUUAUCAACUUGAAAAAGUGGCACCGAGUCGGUGCUUU	[illegible]	-24.7
148	TOP_42_5_3	GGCAGGCGCTCCGCTCGACACGG	chr10	75060621	75060643	minus	75	promoter-TSS	264	NM_001081412	Bcr	protein-coding	GGCAGGCGCUCCGCUCGACA	NO	0.09856516	5.78E-02	0.828105223	GGGCAGGCGCUCCGCUCGACAGUUUUAGAGCUAGAAAUAGCAAGUUAAAAUAAGGCUAGUCCGUUAUCAACUUGAAAAAGUGGCACCGAGUCGGUGCUUU	[illegible]	-26.3
149	TOP_45_2_1	TAGGGTGTATGTATCGCACCAGG	chr5	33213072	33213094	minus	50	TTS	-5155	NM_133903	Spon2	protein-coding	UAGGGUGUAUGUAUCGCACC	YES	0.2412866	1.41E-02	0.172604435	GUAGGGUGUAUGUAUCGCACCGUUUUAGAGCUAGAAAUAGCAAGUUAAAAUAAGGCUAGUCCGUUAUCAACUUGAAAAAGUGGCACCGAGUCGGUGCUUU	[illegible]	-23.5
150	TOP_45_2_2	TAGGGTCTGCGAATACTCCTAGG	chr5	34912696	34912718	minus	50	TTS	3208	NM_207277	Msantd1	protein-coding	UAGGGUCUGCGAAUACUCCU	YES	0.1804432	1.25E-01	0.971578442	GUAGGGUCUGCGAAUACUCCUGUUUUAGAGCUAGAAAUAGCAAGUUAAAAUAAGGCUAGUCCGUUAUCAACUUGAAAAAGUGGCACCGAGUCGGUGCUUU	[illegible]	-24
151	TOP_45_3_1	GTTCATAGTAGGATGCACCCTGG	chr5	108749901	108749923	plus	50	Intergenic	-13930	NR_040691	1700047L14Rik	ncRNA	GUUCAUAGUAGGAUGCACCC	YES	0.07157587	1.53E-01	0.99270789	GGUUCAUAGUAGGAUGCACCCGUUUUAGAGCUAGAAAUAGCAAGUUAAAAUAAGGCUAGUCCGUUAUCAACUUGAAAAAGUGGCACCGAGUCGGUGCUUU	[illegible]	-25.2
152	TOP_45_5_1	CGTGGCCCCCTACGGTTTAGAGG	chr5	108312329	108312351	plus	65	promoter-TSS	-585	NM_001081234	Pigg	protein-coding	CGUGGCCCCCUACGGUUUAG	NO	0.0134043	8.80E-02	0.813268121	GCGUGGCCCCCUACGGUUUAGGUUUUAGAGCUAGAAAUAGCAAGUUAAAAUAAGGCUAGUCCGUUAUCAACUUGAAAAAGUGGCACCGAGUCGGUGCUUU	[illegible]	-23.5
153	TOP_46_5_1	TTTTGCCACCTGTAGTAGTCAGG	chr15	29713550	29713572	plus	45	Intergenic	-459032	NM_008729	Ctnnd2	protein-coding	UUUUGCCACCUGUAGUAGUC	YES	0.3881676	0.093486568	0.987214721	GUUUUGCCACCUGUAGUAGUCGUUUUAGAGCUAGAAAUAGCAAGUUAAAAUAAGGCUAGUCCGUUAUCAACUUGAAAAAGUGGCACCGAGUCGGUGCUUU	[illegible]	-21.6
154	TOP_47_3_1	AAACCGCACTGAACGCAATGGGG	chr13	74062474	74062496	minus	50	promoter-TSS	200	NM_028959	Cep72	protein-coding	AAACCGCACUGAACGCAAUG	YES	0.06996587	1.17E-01	0.829743557	GAAACCGCACUGAACGCAAUGGUUUUAGAGCUAGAAAUAGCAAGUUAAAAUAAGGCUAGUCCGUUAUCAACUUGAAAAAGUGGCACCGAGUCGGUGCUUU	[illegible]	-23
155	TOP_47_5_1	CGCGGGCGATTCGGCAACACAGG	chr13	73937522	73937544	plus	70	promoter-TSS	234	NM_027182	Trip13	protein-coding	CGCGGGCGAUUCGGCAACAC	NO	0.06059476	0.149615212	0.526652785	GCGCGGGCGAUUCGGCAACACGUUUUAGAGCUAGAAAUAGCAAGUUAAAAUAAGGCUAGUCCGUUAUCAACUUGAAAAAGUGGCACCGAGUCGGUGCUUU	[illegible]	-26.5
156	TOP_48_3_1	TCTGGGCTGCTATACTAGGCTGG	chr12	36004130	36004152	minus	55	TTS	-11216	NM_011783	Agr2	protein-coding	UCUGGGCUGCUAUACUAGGC	YES	0.007865912	0.154795232	0.872425779	GUCUGGGCUGCUAUACUAGGCGUUUUAGAGCUAGAAAUAGCAAGUUAAAAUAAGGCUAGUCCGUUAUCAACUUGAAAAAGUGGCACCGAGUCGGUGCUUU	[illegible]	-25.1
157	TOP_48_5_1	TTAAAGACCTATACGGAATAAGG	chr12	33928094	33928116	plus	30	promoter-TSS	-320	NM_033522	Ferd3l	protein-coding	UUAAAGACCUAUACGGAAUA	NO	0.1793309	1.02E-01	0.999920863	GUUAAAGACCUAUACGGAAUAGUUUUAGAGCUAGAAAUAGCAAGUUAAAAUAAGGCUAGUCCGUUAUCAACUUGAAAAAGUGGCACCGAGUCGGUGCUUU	[illegible]	-26.5
158	TOP_49_3_1	CATTGCAAAGGTCCGGTGATAGG	chr6	9248560	9248582	minus	50	TTS	-298552	NM_008751	Nxph1	protein-coding	CAUUGCAAAGGUCCGGUGAU	YES	0.1219576	0.099392663	0.994664223	GCAUUGCAAAGGUCCGGUGAUGUUUUAGAGCUAGAAAUAGCAAGUUAAAAUAAGGCUAGUCCGUUAUCAACUUGAAAAAGUGGCACCGAGUCGGUGCUUU	[illegible]	-23.9
159	TOP_49_5_1	CGTATTGACAGACGTTTAAATGG	chr6	8595406	8595428	plus	35	3' UTR	75359	NM_133236	Glcci1	protein-coding	CGUAUUGACAGACGUUUAAA	NO	0.2156115	1.08E-01	0.987227592	GCGUAUUGACAGACGUUUAAAGUUUUAGAGCUAGAAAUAGCAAGUUAAAAUAAGGCUAGUCCGUUAUCAACUUGAAAAAGUGGCACCGAGUCGGUGCUUU	[illegible]	-23.5
160	TOP_50_3_1	GCACAACCTAGTTAACTGGTTGG	chr10	65003023	65003045	plus	45	3' UTR	-912779	NM_178678	Lrrtm3	protein-coding	GCACAACCUAGUUAACUGGU	YES	0.03585331	1.70E-01	0.933876565	GGCACAACCUAGUUAACUGGUGUUUUAGAGCUAGAAAUAGCAAGUUAAAAUAAGGCUAGUCCGUUAUCAACUUGAAAAAGUGGCACCGAGUCGGUGCUUU	[illegible]	-23.7
161	TOP_50_5_1	GTTACCCTTCAGCGCGAACGTGG	chr10	63429933	63429955	plus	60	promoter-TSS	-154	NM_177612	Ctnna3	protein-coding	GUUACCCUUCAGCGCGAACG	YES	0.09626926	0.091285506	0.808909445	GGUUACCCUUCAGCGCGAACGGUUUUAGAGCUAGAAAUAGCAAGUUAAAAUAAGGCUAGUCCGUUAUCAACUUGAAAAAGUGGCACCGAGUCGGUGCUUU	[illegible]	-22.3
162	TOP_51_3_1	GGTGACGCGCAGGCGACGGCGGG	chr6	121473690	121473712	minus	80	promoter-TSS	23	NM_001033354	Iqsec3	protein-coding	GGUGACGCGCAGGCGACGGC	NO	0.001520125	0.150616271	0.998105517	GGGUGACGCGCAGGCGACGGCGUUUUAGAGCUAGAAAUAGCAAGUUAAAAUAAGGCUAGUCCGUUAUCAACUUGAAAAAGUGGCACCGAGUCGGUGCUUU	[illegible]	-29.1
163	TOP_51_5_1	CGAAAAGCAAGATTCGGCATGGG	chr6	118592084	118592106	plus	45	3' UTR	-29839	NM_001081112	Ankrd26	protein-coding	CGAAAAGCAAGAUUCGGCAU	YES	0.2053154	1.28E-01	0.89317702	GCGAAAAGCAAGAUUCGGCAUGUUUUAGAGCUAGAAAUAGCAAGUUAAAAUAAGGCUAGUCCGUUAUCAACUUGAAAAAGUGGCACCGAGUCGGUGCUUU	[illegible]	-26.1
164	TOP_51_5_2	CAGAGTGAGGACTTACACCGGGG	chr6	118592197	118592219	plus	55	3' UTR	-29952	NM_001081112	Ankrd26	protein-coding	CAGAGUGAGGACUUACACCG	YES	0.03108162	0.122605427	0.999745839	GCAGAGUGAGGACUUACACCGGUUUUAGAGCUAGAAAUAGCAAGUUAAAAUAAGGCUAGUCCGUUAUCAACUUGAAAAAGUGGCACCGAGUCGGUGCUUU	[illegible]	-28.2
165	TOP_52_3_1	TTGTATTTCTAAGACCACAGTGG	chr7	64392423	64392445	plus	35	promoter-TSS	-198	NM_026483	Mphosph10	protein-coding	UUGUAUUUCUAAGACCACAG	NO	0.1260873	1.37E-01	0.999786733	GUUGUAUUUCUAAGACCACAGGUUUUAGAGCUAGAAAUAGCAAGUUAAAAUAAGGCUAGUCCGUUAUCAACUUGAAAAAGUGGCACCGAGUCGGUGCUUU	[illegible]	-26.2
166	TOP_52_5_1	TTAGAGTTCCCCTCCATTGGTGG	chr7	63096958	63096980	minus	50	Intergenic	-115557	NM_007390	Chrna7	protein-coding	UUAGAGUUCCCCUCCAUUGG	YES	0.005967818	8.33E-02	0.993334784	GUUAGAGUUCCCCUCCAUUGGGUUUUAGAGCUAGAAAUAGCAAGUUAAAAUAAGGCUAGUCCGUUAUCAACUUGAAAAAGUGGCACCGAGUCGGUGCUUU	[illegible]	-23.1
167	TOP_53_3_1	AAGCGCTTCTCGTAGACGCCAGG	chr8	115352842	115352864	plus	60	TTS	354041	NM_001025577	Maf	protein-coding	AAGCGCUUCUCGUAGACGCC	YES	0.09240994	2.05E-01	0.940721463	GAAGCGCUUCUCGUAGACGCCGUUUUAGAGCUAGAAAUAGCAAGUUAAAAUAAGGCUAGUCCGUUAUCAACUUGAAAAAGUGGCACCGAGUCGGUGCUUU	[illegible]	-25.6
168	TOP_53_5_1	GCACGTGTAGTCCGGTGTGCTGG	chr8	111448562	111448584	minus	65	TTS	1627	NR_105819	Mir1957b	ncRNA	GCACGUGUAGUCCGGUGUGC	NO	0.1373438	8.49E-02	0.489247901	GGCACGUGUAGUCCGGUGUGCGUUUUAGAGCUAGAAAUAGCAAGUUAAAAUAAGGCUAGUCCGUUAUCAACUUGAAAAAGUGGCACCGAGUCGGUGCUUU	[illegible]	-22.6
169	TOP_54_3_1	GACTATAACTCCAGTTCCGTGGG	chr2	181857587	181857609	plus	45	TTS	-6762	NM_025901	Polr3k	protein-coding	GACUAUAACUCCAGUUCCGU	YES	0.4707997	1.12E-01	0.997149481	GGACUAUAACUCCAGUUCCGUGUUUUAGAGCUAGAAAUAGCAAGUUAAAAUAAGGCUAGUCCGUUAUCAACUUGAAAAAGUGGCACCGAGUCGGUGCUUU	[illegible]	-26.9
170	TOP_54_5_1	GCTCCTTTAAGGCCCGCGTCAGG	chr2	179442342	179442364	plus	65	promoter-TSS	-125	NM_009867	Cdh4	protein-coding	GCUCCUUUAAGGCCCGCGUC	NO	0.08310224	5.33E-02	0.832543157	GGCUCCUUUAAGGCCCGCGUCGUUUUAGAGCUAGAAAUAGCAAGUUAAAAUAAGGCUAGUCCGUUAUCAACUUGAAAAAGUGGCACCGAGUCGGUGCUUU	[illegible]	-24.1
171	TOP_55_3_1	ATCGCTCGCACAGCGATCGAGGG	chr17	86167639	86167661	plus	60	promoter-TSS	-135	NM_011104	Prkce	protein-coding	AUCGCUCGCACAGCGAUCGA	YES	0.05578041	0.004401784	0.145656613	GAUCGCUCGCACAGCGAUCGAGUUUUAGAGCUAGAAAUAGCAAGUUAAAAUAAGGCUAGUCCGUUAUCAACUUGAAAAAGUGGCACCGAGUCGGUGCUUU	[illegible]	-27.6
172	TOP_55_3_2	CATACACCAAAGGGCTCGGTGGG	chr17	86658359	86658381	plus	55	TTS	-95494	NM_010137	Epas1	protein-coding	CAUACACCAAAGGGCUCGGU	YES	0.03200259	1.50E-01	0.956245674	GCAUACACCAAAGGGCUCGGUGUUUUAGAGCUAGAAAUAGCAAGUUAAAAUAAGGCUAGUCCGUUAUCAACUUGAAAAAGUGGCACCGAGUCGGUGCUUU	[illegible]	-26.6
173	TOP_55_5_1	TGGCCTCAGGATGCCCAATTGGG	chr17	85984218	85984240	minus	55	TTS	-160946	NM_030133	Srbd1	protein-coding	UGGCCUCAGGAUGCCCAAUU	YES	0.1287713	0.142297045	0.983645847	GUGGCCUCAGGAUGCCCAAUUGUUUUAGAGCUAGAAAUAGCAAGUUAAAAUAAGGCUAGUCCGUUAUCAACUUGAAAAAGUGGCACCGAGUCGGUGCUUU	[illegible]	-27.3
174	TOP_56_3_1	GTTCCACAAGCAAGCGGTACTGG	chr18	59411075	59411097	plus	55	3' UTR	-168544	NR_105773	Mir6355	ncRNA	GUUCCACAAGCAAGCGGUAC	YES	0.0883117	9.16E-02	0.914207451	GGUUCCACAAGCAAGCGGUACGUUUUAGAGCUAGAAAUAGCAAGUUAAAAUAAGGCUAGUCCGUUAUCAACUUGAAAAAGUGGCACCGAGUCGGUGCUUU	[illegible]	-22.2
175	TOP_56_5_1	ATGGGCGATACAAGTAAAGGTGG	chr18	48045224	48045246	plus	45	promoter-TSS	-100	NM_001025388	Eno1b	protein-coding	AUGGGCGAUACAAGUAAAGG	YES	0.6596843	1.35E-01	0.968225232	GAUGGGCGAUACAAGUAAAGGGUUUUAGAGCUAGAAAUAGCAAGUUAAAAUAAGGCUAGUCCGUUAUCAACUUGAAAAAGUGGCACCGAGUCGGUGCUUU	[illegible]	-24.3
176	TOP_57_3_1	GGAAGGTAGGACTAGCGGGTAGG	chr13	30950602	30950624	plus	60	intron	-2852	NM_153072	Hus1b	protein-coding	GGAAGGUAGGACUAGCGGGU	YES	0.08391187	1.53E-01	0.999953327	GGGAAGGUAGGACUAGCGGGUGUUUUAGAGCUAGAAAUAGCAAGUUAAAAUAAGGCUAGUCCGUUAUCAACUUGAAAAAGUGGCACCGAGUCGGUGCUUU	[illegible]	-34.8
177	TOP_57_5_1	TGTCCGGCCGTGCGGACGTCGGG	chr13	30659969	30659991	minus	75	promoter-TSS	79	NM_134068	Dusp22	protein-coding	UGUCCGGCCGUGCGGACGUC	NO	0.00919198	1.19E-01	0.821144849	GUGUCCGGCCGUGCGGACGUCGUUUUAGAGCUAGAAAUAGCAAGUUAAAAUAAGGCUAGUCCGUUAUCAACUUGAAAAAGUGGCACCGAGUCGGUGCUUU	[illegible]	-27.9
178	TOP_59_3_1	GGTAGAGACCTCTCGTAGGAAGG	chr11	67689130	67689152	minus	55	TTS	6185	NM_009038	Rcvrn	protein-coding	GGUAGAGACCUCUCGUAGGA	YES	0.00663518	8.65E-02	0.680184509	GGGUAGAGACCUCUCGUAGGAGUUUUAGAGCUAGAAAUAGCAAGUUAAAAUAAGGCUAGUCCGUUAUCAACUUGAAAAAGUGGCACCGAGUCGGUGCUUU	[illegible]	-22.2
179	TOP_59_5_1	CTGGCGCAGGCACTCGAATGTGG	chr11	66911903	66911925	minus	65	promoter-TSS	77	NM_178656	Pirt	protein-coding	CUGGCGCAGGCACUCGAAUG	NO	0.1095799	1.01E-01	0.644811585	GCUGGCGCAGGCACUCGAAUGGUUUUAGAGCUAGAAAUAGCAAGUUAAAAUAAGGCUAGUCCGUUAUCAACUUGAAAAAGUGGCACCGAGUCGGUGCUUU	[illegible]	-22.9
180	TOP_60_5_1	CGGCTAGGTTTCCCACACGGAGG	chr2	151494140	151494162	plus	65	promoter-TSS	-31	NM_198326	Nsfl1c	protein-coding	CGGCUAGGUUUCCCACACGG	NO	0.009084254	1.39E-01	0.997002374	GCGGCUAGGUUUCCCACACGGGUUUUAGAGCUAGAAAUAGCAAGUUAAAAUAAGGCUAGUCCGUUAUCAACUUGAAAAAGUGGCACCGAGUCGGUGCUUU	[illegible]	-28.3
181	TOP_61_3_1	CTAGAGGCAAGGTAGACGGCTGG	chr15	81015312	81015334	plus	60	intron	37558	NM_134091	Sgsm3	protein-coding	CUAGAGGCAAGGUAGACGGC	YES	0.1717171	0.112979136	0.95415964	GCUAGAGGCAAGGUAGACGGCGUUUUAGAGCUAGAAAUAGCAAGUUAAAAUAAGGCUAGUCCGUUAUCAACUUGAAAAAGUGGCACCGAGUCGGUGCUUU	[illegible]	-24.5
182	TOP_63_3_1	CTAGCTATACGGTCCGGACCTGG	chr3	46448142	46448164	minus	60	promoter-TSS	212	NM_001101479	Pabpc4l	protein-coding	CUAGCUAUACGGUCCGGACC	YES	0.005785776	1.03E-01	0.982486494	GCUAGCUAUACGGUCCGGACCGUUUUAGAGCUAGAAAUAGCAAGUUAAAAUAAGGCUAGUCCGUUAUCAACUUGAAAAAGUGGCACCGAGUCGGUGCUUU	[illegible]	-28.7
183	TOP_64_3_1	CAGGCGGGCCACTCGCGCCTCGG	chr17	34837050	34837072	minus	80	promoter-TSS	-42	NM_033613	Dxo	protein-coding	CAGGCGGGCCACUCGCGCCU	NO	0.02881063	1.34E-01	0.956683936	GCAGGCGGGCCACUCGCGCCUGUUUUAGAGCUAGAAAUAGCAAGUUAAAAUAAGGCUAGUCCGUUAUCAACUUGAAAAAGUGGCACCGAGUCGGUGCUUU	[illegible]	-31
184	TOP_64_5_1	TGGAGCATAGTATCGGTGAGAGG	chr17	34670286	34670308	plus	50	promoter-TSS	-238	NM_031176	Tnxb	protein-coding	UGGAGCAUAGUAUCGGUGAG	YES	0.3466373	1.66E-01	0.993588357	GUGGAGCAUAGUAUCGGUGAGGUUUUAGAGCUAGAAAUAGCAAGUUAAAAUAAGGCUAGUCCGUUAUCAACUUGAAAAAGUGGCACCGAGUCGGUGCUUU	[illegible]	-23.8
185	TOP_66_3_1	CCAGGATATCACTAGTTAGTGGG	chr12	41449381	41449403	plus	40	intron	36665	NM_010733	Lrrn3	protein-coding	CCAGGAUAUCACUAGUUAGU	YES	0.07187974	1.50E-01	0.999648237	GCCAGGAUAUCACUAGUUAGUGUUUUAGAGCUAGAAAUAGCAAGUUAAAAUAAGGCUAGUCCGUUAUCAACUUGAAAAAGUGGCACCGAGUCGGUGCUUU	[illegible]	-27.4
186	TOP_66_3_2	ATCATGAGAGGTTTCGTGAAAGG	chr12	41450150	41450172	minus	40	intron	-35896	NM_010733	Lrrn3	protein-coding	AUCAUGAGAGGUUUCGUGAA	YES	0.1924366	1.44E-02	0.141747906	GAUCAUGAGAGGUUUCGUGAAGUUUUAGAGCUAGAAAUAGCAAGUUAAAAUAAGGCUAGUCCGUUAUCAACUUGAAAAAGUGGCACCGAGUCGGUGCUUU	[illegible]	-23.2
187	TOP_66_5_1	GAATACAATACTACTAGCTGTGG	chr12	40175289	40175311	plus	35	Intergenic	24015	NM_001033437	Lsmem1	protein-coding	GAAUACAAUACUACUAGCUG	NO	0.3808325	0.105068246	0.997326921	GGAAUACAAUACUACUAGCUGGUUUUAGAGCUAGAAAUAGCAAGUUAAAAUAAGGCUAGUCCGUUAUCAACUUGAAAAAGUGGCACCGAGUCGGUGCUUU	[illegible]	-27.8
188	TOP_66_5_2	TATACTGTACATACAGACGGAGG	chr12	40175349	40175371	minus	40	Intergenic	-23955	NM_001033437	Lsmem1	protein-coding	UAUACUGUACAUACAGACGG	YES	0.2766103	6.33E-02	0.962733617	GUAUACUGUACAUACAGACGGGUUUUAGAGCUAGAAAUAGCAAGUUAAAAUAAGGCUAGUCCGUUAUCAACUUGAAAAAGUGGCACCGAGUCGGUGCUUU	[illegible]	-22.6
189	TOP_66_5_3	GCTCTTCCTTCGGTGATCGCTGG	chr12	40176601	40176623	minus	60	3' UTR	-22703	NM_001033437	Lsmem1	protein-coding	GCUCUUCCUUCGGUGAUCGC	YES	0.3032185	1.22E-01	0.643727215	GGCUCUUCCUUCGGUGAUCGCGUUUUAGAGCUAGAAAUAGCAAGUUAAAAUAAGGCUAGUCCGUUAUCAACUUGAAAAAGUGGCACCGAGUCGGUGCUUU	[illegible]	-24
190	TOP_67_5_1	AATCTTACACTAACCCTACCTGG	chr2	104027591	104027613	minus	40	promoter-TSS	197	NM_020593	Fbxo3	protein-coding	AAUCUUACACUAACCCUACC	YES	0.7041557	9.88E-02	0.984652088	GAAUCUUACACUAACCCUACCGUUUUAGAGCUAGAAAUAGCAAGUUAAAAUAAGGCUAGUCCGUUAUCAACUUGAAAAAGUGGCACCGAGUCGGUGCUUU	[illegible]	-21.6
191	TOP_68_5_1	AGAGCGTCGGACGGAAGCGTGGG	chr15	90224270	90224292	minus	65	promoter-TSS	30	NM_001033441	Alg10b	protein-coding	AGAGCGUCGGACGGAAGCGU	NO	0.01008914	0.134318057	0.999936591	GAGAGCGUCGGACGGAAGCGUGUUUUAGAGCUAGAAAUAGCAAGUUAAAAUAAGGCUAGUCCGUUAUCAACUUGAAAAAGUGGCACCGAGUCGGUGCUUU	[illegible]	-31.2
192	TOP_70_5_1	TTATAATTCAGTTCTCGAGTAGG	chr12	46693893	46693915	plus	30	TTS	124871	NM_021361	Nova1	protein-coding	UUAUAAUUCAGUUCUCGAGU	NO	0.2649453	1.66E-01	0.976086094	GUUAUAAUUCAGUUCUCGAGUGUUUUAGAGCUAGAAAUAGCAAGUUAAAAUAAGGCUAGUCCGUUAUCAACUUGAAAAAGUGGCACCGAGUCGGUGCUUU	[illegible]	-21.5
193	TOP_71_5_1	CATTATGGCGGCACGCCGACCGG	chr2	112239397	112239419	plus	65	promoter-TSS	-433	NM_207206	Lpcat4	protein-coding	CAUUAUGGCGGCACGCCGAC	NO	0.0022599	1.40E-01	0.999760034	GCAUUAUGGCGGCACGCCGACGUUUUAGAGCUAGAAAUAGCAAGUUAAAAUAAGGCUAGUCCGUUAUCAACUUGAAAAAGUGGCACCGAGUCGGUGCUUU	[illegible]	-30.5
194	TOP_72_2_1	GGTGCATCACTATATGGGGCGGG	chr7	65644821	65644843	minus	55	promoter-TSS	66	NM_172310	Tarsl2	protein-coding	GGUGCAUCACUAUAUGGGGC	YES	0.2337856	8.08E-02	0.937148321	GGGUGCAUCACUAUAUGGGGCGUUUUAGAGCUAGAAAUAGCAAGUUAAAAUAAGGCUAGUCCGUUAUCAACUUGAAAAAGUGGCACCGAGUCGGUGCUUU	[illegible]	-23
195	TOP_74_3_1	ATGGGATGGATCCCCGGATATGG	chr1	6563296	6563318	plus	55	intron	76076	NM_173868	St18	protein-coding	AUGGGAUGGAUCCCCGGAUA	YES	0.1149611	8.20E-02	0.999745468	GAUGGGAUGGAUCCCCGGAUAGUUUUAGAGCUAGAAAUAGCAAGUUAAAAUAAGGCUAGUCCGUUAUCAACUUGAAAAAGUGGCACCGAGUCGGUGCUUU	[illegible]	-28.8
196	TOP_76_3_1	TGTGAATCTCTGCCGCTCTTGGG	chr4	102971230	102971252	plus	50	3' UTR	-15138	NM_026100	Tctex1d1	protein-coding	UGUGAAUCUCUGCCGCUCUU	YES	0.404061	1.31E-01	0.96874605	GUGUGAAUCUCUGCCGCUCUUGUUUUAGAGCUAGAAAUAGCAAGUUAAAAUAAGGCUAGUCCGUUAUCAACUUGAAAAAGUGGCACCGAGUCGGUGCUUU	[illegible]	-26
197	TOP_76_5_1	CTATTTAGCCACTACAGGGGAGG	chr4	97182383	97182405	plus	50	Intergenic	-397234	NM_001081284	Gm12695	protein-coding	CUAUUUAGCCACUACAGGGG	YES	0.0174336	0.105996434	0.708772905	GCUAUUUAGCCACUACAGGGGGUUUUAGAGCUAGAAAUAGCAAGUUAAAAUAAGGCUAGUCCGUUAUCAACUUGAAAAAGUGGCACCGAGUCGGUGCUUU	[illegible]	-23
198	TOP_77_5_1	GTTCAAAACGTAAACACCACTGG	chr1	54984864	54984886	plus	40	TTS	42603	NM_031179	Sf3b1	protein-coding	GUUCAAAACGUAAACACCAC	YES	0.0976016	1.24E-01	0.996485788	GGUUCAAAACGUAAACACCACGUUUUAGAGCUAGAAAUAGCAAGUUAAAAUAAGGCUAGUCCGUUAUCAACUUGAAAAAGUGGCACCGAGUCGGUGCUUU	[illegible]	-25.5
199	TOP_78_3_1	TAGGTAGATCTTTGAGTTCGAGG	chr4	132887197	132887219	minus	40	Intergenic	2750	NM_133887	Stx12	protein-coding	UAGGUAGAUCUUUGAGUUCG	YES	0.3295554	1.17E-01	0.843985775	GUAGGUAGAUCUUUGAGUUCGGUUUUAGAGCUAGAAAUAGCAAGUUAAAAUAAGGCUAGUCCGUUAUCAACUUGAAAAAGUGGCACCGAGUCGGUGCUUU	[illegible]	-22.3
200	TOP_78_5_1	TGTTTTGTAGACATGTCCGCTGG	chr6	92772107	92772129	minus	45	TTS	65934	NM_001081146	Prickle2	protein-coding	UGUUUUGUAGACAUGUCCGC	YES	0.1991074	8.33E-02	0.985206037	GUGUUUUGUAGACAUGUCCGCGUUUUAGAGCUAGAAAUAGCAAGUUAAAAUAAGGCUAGUCCGUUAUCAACUUGAAAAAGUGGCACCGAGUCGGUGCUUU	[illegible]	-23.2
201	TOP_78_5_2	TAAAGTGTGGCTCGCTGTGCCGG	chr6	95473827	95473849	plus	55	3' UTR	-139845	NR_033559	AY512915	ncRNA	UAAAGUGUGGCUCGCUGUGC	YES	0.02506806	1.31E-01	0.875583141	GUAAAGUGUGGCUCGCUGUGCGUUUUAGAGCUAGAAAUAGCAAGUUAAAAUAAGGCUAGUCCGUUAUCAACUUGAAAAAGUGGCACCGAGUCGGUGCUUU	[illegible]	-25
202	TOP_80_3_1	TGAGCAGCGTAGATAGGCTTAGG	chr6	108860125	108860147	plus	50	TTS	31495	NM_138677	Edem1	protein-coding	UGAGCAGCGUAGAUAGGCUU	YES	0.06400072	0.168350498	0.999150718	GUGAGCAGCGUAGAUAGGCUUGUUUUAGAGCUAGAAAUAGCAAGUUAAAAUAAGGCUAGUCCGUUAUCAACUUGAAAAAGUGGCACCGAGUCGGUGCUUU	[illegible]	-32.2
203	TOP_81_3_1	ATAGAGCACCGTTGCCTAATTGG	chr5	67438424	67438446	plus	45	Intergenic	-10636	NM_001164806	Bend4	protein-coding	AUAGAGCACCGUUGCCUAAU	YES	0.05172534	1.59E-01	0.998208009	GAUAGAGCACCGUUGCCUAAUGUUUUAGAGCUAGAAAUAGCAAGUUAAAAUAAGGCUAGUCCGUUAUCAACUUGAAAAAGUGGCACCGAGUCGGUGCUUU	[illegible]	-27.3
204	TOP_81_5_1	GAGCTGGACCTCACCGCGCGGGG	chr5	55624025	55624047	plus	75	Intergenic	1274044	NM_177871	Gm10440	ncRNA	GAGCUGGACCUCACCGCGCG	NO	0.005763679	1.37E-01	0.99129224	GGAGCUGGACCUCACCGCGCGGUUUUAGAGCUAGAAAUAGCAAGUUAAAAUAAGGCUAGUCCGUUAUCAACUUGAAAAAGUGGCACCGAGUCGGUGCUUU	[illegible]	-26.8
205	TOP_82_3_1	ACAGGTGTTCCGATGTGTAGTGG	chr6	42142117	42142139	minus	50	TTS	-1192	NM_181275	Tas2r139	protein-coding	ACAGGUGUUCCGAUGUGUAG	YES	0.126586	1.29E-01	0.956487682	GACAGGUGUUCCGAUGUGUAGGUUUUAGAGCUAGAAAUAGCAAGUUAAAAUAAGGCUAGUCCGUUAUCAACUUGAAAAAGUGGCACCGAGUCGGUGCUUU	[illegible]	-23.2
206	TOP_82_5_1	CTCCTGGGGGGCCGCGGTATTGG	chr6	41846753	41846775	minus	75	promoter-TSS	785	NM_008843	Pip	protein-coding	CUCCUGGGGGGCCGCGGUAU	NO	0.1141952	9.52E-02	0.91591696	GCUCCUGGGGGGCCGCGGUAUGUUUUAGAGCUAGAAAUAGCAAGUUAAAAUAAGGCUAGUCCGUUAUCAACUUGAAAAAGUGGCACCGAGUCGGUGCUUU	[illegible]	-27.7
207	TOP_83_3_1	GGAGCATGGCATGCTGCGCCTGG	chr15	76906380	76906402	plus	70	TTS	2320	NM_012053	Rpl8	protein-coding	GGAGCAUGGCAUGCUGCGCC	NO	0.007149751	1.33E-01	0.992927521	GGGAGCAUGGCAUGCUGCGCCGUUUUAGAGCUAGAAAUAGCAAGUUAAAAUAAGGCUAGUCCGUUAUCAACUUGAAAAAGUGGCACCGAGUCGGUGCUUU	[illegible]	-28.6
208	TOP_83_3_2	CGGTACTGACGTAAATTATGTGG	chr15	76906520	76906542	plus	40	TTS	2460	NM_012053	Rpl8	protein-coding	CGGUACUGACGUAAAUUAUG	YES	0.2981161	0.114093066	0.949143074	GCGGUACUGACGUAAAUUAUGGUUUUAGAGCUAGAAAUAGCAAGUUAAAAUAAGGCUAGUCCGUUAUCAACUUGAAAAAGUGGCACCGAGUCGGUGCUUU	[illegible]	-23.2
209	TOP_83_3_3	GGGGAAGAAGGTCCTAACGATGG	chr15	78596826	78596848	minus	55	promoter-TSS	210	NM_028195	Cyth4	protein-coding	GGGGAAGAAGGUCCUAACGA	YES	0.07818274	8.78E-02	0.799817067	GGGGGAAGAAGGUCCUAACGAGUUUUAGAGCUAGAAAUAGCAAGUUAAAAUAAGGCUAGUCCGUUAUCAACUUGAAAAAGUGGCACCGAGUCGGUGCUUU	[illegible]	-22.5

210	TOP_83_5_1	TATGCAATGGTGTCAGCGTTTGG	chr1	3065249	3065271	plus	45	Intergenic	606238	NM_001011874	Xkr4	protein-coding	UAUGCAAUGGUGUCAGCGUU	YES	0.1929498	1.36E-01	0.999837686	GUAUGCAAUGGUGUCAGCGUUGUUUUAGAGCUAGAAAUAGCAAGUUAAAAUAAGGCUAGUCCGUUAUCAACUUGAAAAAGUGGCACCGAGUCGGUGCUUU	......(((((....(((.(((((((((.((((....))))...))))))))).)))...)))))....((((....))))(((((((...)))))))..	-30.5
211	TOP_83_5_2	CTGATTATGGGGTGGATCCCTGG	chr1	3137051	3137073	plus	55	Intergenic	534436	NM_001011874	Xkr4	protein-coding	CUGAUUAUGGGGUGGAUCCC	YES	0.000160592	0.170083569	0.999634315	GCUGAUUAUGGGGUGGAUCCCGUUUUAGAGCUAGAAAUAGCAAGUUAAAAUAAGGCUAGUCCGUUAUCAACUUGAAAAAGUGGCACCGAGUCGGUGCUUU	..((((.(((((.(((.((..(((((((.((((....))))...)))))))..))))).))))).))))((((....))))(((((((...)))))))..	-31.3
212	TOP_84_3_1	AATTCAGCTCGGTTAGGCATGGG	chr12	88851008	88851030	plus	45	intron	56515	NM_172544	Nrxn3	protein-coding	AAUUCAGCUCGGUUAGGCAU	YES	0.1520075	1.19E-01	0.998906279	GAAUUCAGCUCGGUUAGGCAUGUUUUAGAGCUAGAAAUAGCAAGUUAAAAUAAGGCUAGUCCGUUAUCAACUUGAAAAAGUGGCACCGAGUCGGUGCUUU	..........(((...(((.((((((((.((((....))))...))))))))..)))...)))......((((....))))(((((((...)))))))..	-26.1
213	TOP_84_3_2	ATCAGATCAAGTAACCGTCCAGG	chr12	88851418	88851440	plus	45	intron	56925	NM_172544	Nrxn3	protein-coding	AUCAGAUCAAGUAACCGUCC	YES	0.7748023	1.13E-01	0.815636997	GAUCAGAUCAAGUAACCGUCCGUUUUAGAGCUAGAAAUAGCAAGUUAAAAUAAGGCUAGUCCGUUAUCAACUUGAAAAAGUGGCACCGAGUCGGUGCUUU	.......(((((((((.(((.(((((((.((((....))))...)))))))..)))......)))....))))))......(((((((...)))))))..	-19.9
214	TOP_84_3_3	GGACTCCATAAATTCCGAGATGG	chr12	88851547	88851569	plus	45	intron	57054	NM_172544	Nrxn3	protein-coding	GGACUCCAUAAAUUCCGAGA	YES	0.1142214	1.09E-01	0.95308496	GGGACUCCAUAAAUUCCGAGAGUUUUAGAGCUAGAAAUAGCAAGUUAAAAUAAGGCUAGUCCGUUAUCAACUUGAAAAAGUGGCACCGAGUCGGUGCUUU	.(((((((..((((((...))))))....((((....))))............))..)))))......((((....))))(((((((...)))))))..	-23
215	TOP_84_5_1	GCTAGCACGAGCTATCTAGGAGG	chr12	73960974	73960996	plus	55	Intergenic	-3545	NM_178392	Snapc1	protein-coding	GCUAGCACGAGCUAUCUAGG	YES	0.01920792	2.17E-01	0.998941016	GGCUAGCACGAGCUAUCUAGGGUUUUAGAGCUAGAAAUAGCAAGUUAAAAUAAGGCUAGUCCGUUAUCAACUUGAAAAAGUGGCACCGAGUCGGUGCUUU	(((((((((..(((((((((..........)))))..))))..))........)))))))........((((....))))(((((((...)))))))..	-28.8
216	TOP_84_5_2	ATTGGCCGTGTGAGTAGACTGGG	chr12	83793586	83793608	plus	50	TTS	29963	NM_130887	Papln	protein-coding	AUUGGCCGUGUGAGUAGACU	YES	0.2310749	0.12827587	0.999276439	GAUUGGCCGUGUGAGUAGACUGUUUUAGAGCUAGAAAUAGCAAGUUAAAAUAAGGCUAGUCCGUUAUCAACUUGAAAAAGUGGCACCGAGUCGGUGCUUU	((((((((((........))((((((((.((((....))))...)))))))).))))))))........((((....))))(((((((...)))))))..	-28.1
217	TOP_85_3_1	GCTCTAACAATCAACGAGTTAGG	chr7	120956772	120956794	plus	40	TTS	25529	NM_007672	Cdr2	protein-coding	GCUCUAACAAUCAACGAGUU	YES	0.1018974	0.11098882	0.999643471	GGCUCUAACAAUCAACGAGUUGUUUUAGAGCUAGAAAUAGCAAGUUAAAAUAAGGCUAGUCCGUUAUCAACUUGAAAAAGUGGCACCGAGUCGGUGCUUU	(((((((((((((......)))))..))))))))........((((((...((((((.....)).)))).))))))......(((((((...)))))))..	-26.8
218	TOP_85_5_1	GGTCACCAAGTTCGCGGTACCGG	chr7	120634868	120634890	plus	60	promoter-TSS	-310	NM_025899	Uqcrc2	protein-coding	GGUCACCAAGUUCGCGGUAC	YES	0.1884745	1.29E-01	0.972333211	GGGUCACCAAGUUCGCGGUACGUUUUAGAGCUAGAAAUAGCAAGUUAAAAUAAGGCUAGUCCGUUAUCAACUUGAAAAAGUGGCACCGAGUCGGUGCUUU	.......((((((.((((.(((((((((.((((....))))...))))))).......)))))....))))))......(((((((...)))))))..	-23.9

Supplementary Table 2: GC percentages in Surveyor positve and negative sequences

	**Surveyor Positive** GC (%) ± SD	**Surveyor Negative** GC (%) ± SD	***P* value[1]**	***P* value[2]**	***P* value[3]**
Pos 1 - 20	54.03 ± 10.95	53.6 ± 11.94	0.785	0.895	0.780
Pos 1-6	51.03 ± 18.25	47.57 ± 19.06	0.181	0.222	0.587
Pos 7-14	53.29 ± 18.26	55.9 ± 18.76	0.31	0.997	0.306
Pos 15-20	58.01 ± 19.88	56.55 ± 18.91	0.585	0.531	0.177

P value[1]: Welch Two Sample T test *P* value; *P* value[2] : Non parametric Kolmogorov-Smirnov test *P* value
P value[3] : logistic regression *P* value

Supplementary Table 3: Complete results of logistic regression analysis

	Estimate	Std. Error	Z	*P* value	
(Intercept)	1.897	4.422	0.429	0.6679	
Pos_1_A	-0.043	0.740	-0.058	0.9535	
Pos_1_G	0.054	0.806	0.066	0.9471	
Pos_1_T	-0.391	0.942	-0.415	0.6782	
Pos_2_C	0.107	0.822	0.130	0.8963	
Pos_2_G	-0.996	0.814	-1.223	0.2212	
Pos_2_T	-2.419	0.900	-2.688	0.0072	**
Pos_3_C	-1.027	0.830	-1.238	0.2158	
Pos_3_G	-2.464	0.847	-2.911	0.0036	**
Pos_3_T	-0.048	0.722	-0.066	0.9471	
Pos_4_A	-0.858	0.840	-1.021	0.3070	
Pos_4_G	-0.342	0.839	-0.407	0.6837	
Pos_4_T	-1.366	0.896	-1.525	0.1273	
Pos_5_A	0.597	0.771	0.774	0.4388	
Pos_5_G	1.660	0.897	1.851	0.0642	.
Pos_5_T	-0.077	0.725	-0.106	0.9157	
Pos_6_A	-2.049	0.898	-2.281	0.0225	*
Pos_6_G	-0.801	0.869	-0.921	0.3570	
Pos_6_T	1.599	0.856	1.869	0.0616	.
Pos_7_A	0.537	0.841	0.639	0.5231	
Pos_7_G	1.185	0.797	1.486	0.1372	
Pos_7_T	1.194	0.816	1.463	0.1435	
Pos_8_A	0.084	0.881	0.096	0.9239	
Pos_8_G	-2.110	0.804	-2.625	0.0087	**
Pos_8_T	-1.368	0.819	-1.671	0.0948	.
Pos_9_A	-0.301	0.901	-0.334	0.7385	
Pos_9_C	-1.049	0.806	-1.302	0.1929	
Pos_9_G	-0.914	0.856	-1.068	0.2855	
Pos_10_C	-0.217	0.837	-0.259	0.7957	
Pos_10_G	0.771	0.795	0.970	0.3321	
Pos_10_T	1.426	0.868	1.643	0.1004	
Pos_11_A	0.687	0.816	0.842	0.4000	
Pos_11_G	-1.861	0.854	-2.178	0.0294	*
Pos_11_T	-1.397	0.830	-1.682	0.0926	.
Pos_12_A	-0.347	0.869	-0.399	0.6900	
Pos_12_G	-0.941	0.815	-1.155	0.2482	
Pos_12_T	-1.634	0.889	-1.838	0.0661	.
Pos_13_A	-0.182	0.863	-0.211	0.8329	
Pos_13_G	-0.599	0.737	-0.813	0.4164	
Pos_13_T	-0.238	0.808	-0.295	0.7678	
Pos_14_A	2.535	1.007	2.518	0.0118	*
Pos_14_C	0.954	0.848	1.126	0.2603	
Pos_14_G	-0.167	0.858	-0.195	0.8455	
Pos_15_A	-0.179	0.885	-0.202	0.8400	
Pos_15_G	-1.196	0.785	-1.523	0.1277	
Pos_15_T	0.391	0.825	0.473	0.6360	
Pos_16_A	0.540	0.890	0.607	0.5440	
Pos_16_G	-0.364	0.728	-0.500	0.6168	
Pos_16_T	-0.310	0.747	-0.415	0.6783	
Pos_17_A	-0.423	0.864	-0.490	0.6245	
Pos_17_G	-2.419	0.814	-2.970	0.0030	**
Pos_17_T	-1.489	0.863	-1.725	0.0846	.
Pos_18_A	1.220	0.837	1.457	0.1451	
Pos_18_G	-1.798	0.816	-2.205	0.0275	*
Pos_18_T	-1.252	0.888	-1.411	0.1583	
Pos_19_A	1.785	0.768	2.326	0.0200	*
Pos_19_C	1.827	0.891	2.051	0.0403	*
Pos_19_T	1.350	1.024	1.317	0.1877	
Pos_20_A	-2.216	0.959	-2.311	0.0208	*
Pos_20_C	0.036	0.695	0.051	0.9593	
Pos_20_G	-1.324	0.873	-1.516	0.1294	
Pos_21_C	-0.708	1.279	-0.554	0.5797	
Pos_21_G	-0.714	0.824	-0.866	0.3863	
Pos_21_T	1.120	0.776	1.443	0.1490	
3_UTR	1.157	1.172	0.987	0.3236	
Context_promoter-TSS	3.862	1.256	3.075	0.0021	**
Context_TTS	0.446	0.878	0.508	0.6114	
Context_5'	21.480	1184.000	0.018	0.9855	
Context_intron	-1.378	1.266	-1.089	0.2764	
Context_exon	-1.463	1.664	-0.879	0.3792	
MFE	-0.010	0.147	-0.066	0.9470	
unpairing_probability of_guide_sequence	-9.054	2.505	-3.614	0.0003	***
ave_tracer_impact	6.144	8.261	0.744	0.4570	
max_tracer_impact	0.018	1.969	0.009	0.9926	
NormalGCYES	3.143	0.950	3.311	0.0009	***
Strand (plus)	0.624	0.605	1.031	0.3027	

UTR: untranslated region; TTS: transcription termination site; TSS: transcription start site

Asterisk indicates significant; dot indicates marginal significance
